# Unrevealed effect of silver species on hydrocarbon pool in ethylene conversion on ZSM-5 by 2D COS FT-IR and 2D COS UV-vis *operando* spectroscopies

**DOI:** 10.1016/j.isci.2025.113650

**Published:** 2025-09-25

**Authors:** Karolina A. Tarach, Agata Kordek, Oliwia Rogala, Gabriela Jajko-Liberka, Małgorzata Smoliło-Utrata, Anna Walczyk, Joaquin Martinez-Triguero, Fernando Rey, Kinga Góra-Marek

**Affiliations:** 1 Faculty of Chemistry, Jagiellonian University in Kraków, Gronostajowa 2, 30-387 Kraków, Poland; 2 Doctoral School of Exact and Natural Sciences, Jagiellonian University in Kraków, Łojasiewicza 11, 30-348 Kraków, Poland; 3 Instituto de Tecnologia Química, Universitat Politècnica de València – Consejo Superior de Investigaciones Científicas (UPV-CSIC), Avda. de los Naranjos s/n, 46022 Valencia, Spain

**Keywords:** Catalysis, Materials synthesis, Synthetic inorganic chemistry

## Abstract

Silver-loaded and protonic ZSM-5 zeolites were investigated for the transformation of ethylene into propylene and aromatics. *Operando* FT-IR and UV-vis spectroscopic studies provided unique insights into the influence of silver species in ZSM-5 on ethylene conversion pathways. Acetone washing altered the distribution of silver on the ion-exchanged zeolite surface. *Operando* spectroscopic studies and catalytic tests (350°C) revealed that catalytic performance strongly depended on the method of zeolite treatment. The crucial role of silver speciation is suggested in the participation of silver sites in ethylene conversion. The high stability of the acetone-washed silver-ZSM-5 sample was confirmed by analysis of coke species. Two-dimensional correlation spectroscopy (2D COS) allowed the identification of alkyl-substituted benzenium and cyclopentenyl cations as important intermediates in ethylene transformation, indicating similarities with the hydrocarbon pool (HP) mechanism. To date, the heterospectral 2D COS analysis has not been performed on *operando* spectroscopic results of heterogeneous catalytic studies; in this work, we present its application.

## Highlights

• *Operando* FT-IR and UV-vis spectroscopic studies with 2D correlation analysis
• Silver-loaded ZSM-5 zeolite as an efficient ethylene oligomerization catalyst
• Silver speciation modified by the acetone wash is crucial for the ethylene oligomerization
• Similar spectroscopic features of ethylene conversion and hydrocarbon pool species

## Graphical abstract

**Figure:**
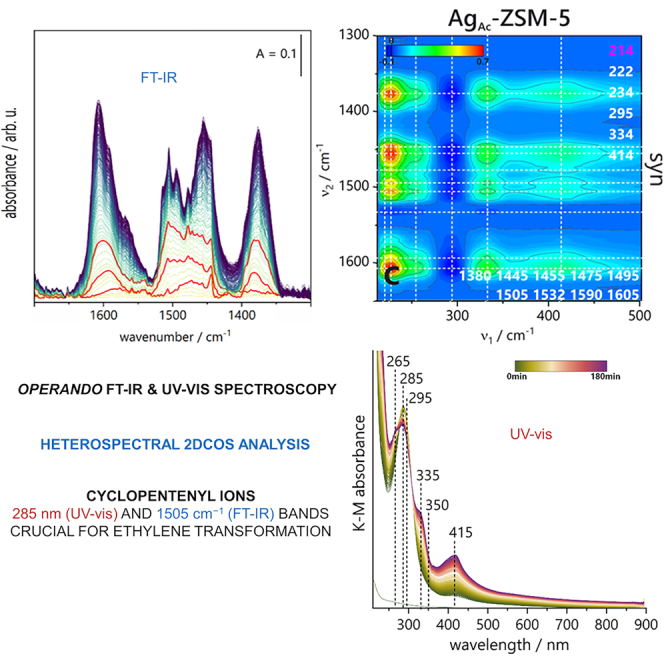

## Introduction

Light olefins are crucial raw materials in numerous petrochemical processes. Ethylene, e.g., produced from bioethanol, can be used in ethylene-to-propene conversion (ETP) employing zeolites as catalysts. The main factor ruling the shape-selective action of zeolites is a steric effect, i.e., only molecules with a kinetic diameter smaller than the pore opening dimensions are allowed to enter the pores and to react on an active site, or to leave them as the products of the reaction. The shape selectivity effect also restricts the formation of bulky transition state intermediates within the pores, thereby preventing the formation of undesired products. The acidic properties of zeolites, concerning the quantity of Brønsted (H^+^) and Lewis acid sites (e.g., Na^+^, Ag^+^), also significantly affect alkene transformation. Several zeolites and molecular sieves with different framework topologies and chemical compositions have been tested as ETP catalysts to date. Among them, the protonic form (H-SSZ-13) of the small-pore zeolite SSZ-13 (framework type CHA) has been reported to provide a high propene selectivity.^1^^,^^2^^,^^3^^,^^4^^,^^5^

While the Hydrocarbon Pool (HCP) mechanism is most well-known for MTO, MTH, MTG, and MTA (methanol-to-olefins, -hydrocarbons, -gasoline, -aromatics, respectively) processes, recent studies have documented its involvement in other reactions as well, especially those involving C_1_ or small oxygenates converted over zeolite catalysts. Cyclopentenyl cations (five-membered ring enyl cations, alternative names taken from Hernandez, Jentoft^6^) are among the crucial species found in the hydrocarbon pool. They are possible intermediates in the side-chain alkylation and pairing pathways, wherein light olefins are extracted from cyclic ions. An increased concentration of the cyclopentenyl cations accelerates the reaction.^7^ Solid-state NMR spectroscopy and GC-MS analysis have demonstrated that cyclopentenyl cations serve as active intermediates in the propane aromatization on Ga/ZSM-5 zeolite. In this process, cyclopentenyl cations serve as essential hydrocarbon pool species that co-catalyze propane conversion and facilitate aromatics production, which highlights the significance of the hydrocarbon pool mechanism in propane aromatization.^8^ The hydrocarbon pool mechanism of zeolite-catalyzed ethene to propene (ETP) conversion has been thoroughly investigated using H-UZM-35 zeolite.^9^ A bicyclic aromatic-based mechanism involving naphthalene, C-2 substituted bicyclic aromatics, and 2-isopropyl-7-methylnaphthalene as hydrocarbon pool species has been proposed for the zeolite-catalyzed ETP reaction.

Nanoparticles and silver ions within the zeolite framework have been shown to enhance ethylene conversion by enhancing alkene adsorption capacity, thus increasing ethylene conversion. Yang et al. have validated the existence of energetically favourable interactions among ethylene molecules and silver clusters.^10^ The Ag^+^ cations facilitated the formation of π-complexes with ethylene, inhibiting alkene oligomerization at Brønsted acid sites at low temperatures. Furthermore, in the presence of Ag^+^-Lewis acid sites, at temperatures above 350°C, ethylene was converted into aromatics.^11^ Hsieh et al.^12^ have recognized Ag-ZSM-5 as an efficient catalyst for low-temperature ethylene-to-liquid conversions. By employing DFT calculations, they indicated that the binding energy of ethylene on Ag^+^ sites is more favourable (i.e., exhibits a lower enthalpy of adsorption). In contrast, the activation barrier for ethylene is reduced on H^+^ sites. While the Ag^+^ cations coordinate ethylene molecules to form π-complexes, the protons associated with the zeolite framework, i.e., Brønsted acid sites, facilitate the initial oligomerization of ethylene, resulting in longer-chain hydrocarbons. These reactions are part of the hydrocarbon pool mechanism, classified as the “alkene cycle.”

In this work, cyclopentenyl cations are observed in ethylene oligomerization on Ag-ZSM-5 by cross-identifying FT-IR and UV-vis bands using heterospectral 2D COS, an approach not previously reported in the literature. These cyclic carbocations exhibit high reactivity in the ring-expansion to form aromatics. The crucial role of silver speciation is revealed in the formation of cyclopentenyl intermediates. The experimental results provide strong evidence for the diverse dispersion of silver species, with a predominant presence of Ag_3_^+^ in the Ag_Ac_-ZSM-5 sample, which resulted in highly stable (16 h) activity and selectivity during ethylene conversion at 350°C. The spent Ag_Ac_-ZSM-5 catalyst is rich in highly alkyl-substituted aromatics, with a lower total carbonaceous deposit content compared to protonic H-ZSM-5. Comprehensive insight from FT-IR and UV-vis *operando* investigations provided an understanding of the nature of the species actively participating in the catalytic cycle. This knowledge is helpful in evaluating the impact of silver status on the conversion process, thereby contributing to the development of efficient catalysts for the synthesis of propene with high selectivity.

## Results and discussion

### The nature of silver sites assessed with *in situ* spectroscopic investigations

The Ag-ZSM-5 catalysts (2–2.5 wt. % Ag loading) were prepared by the ion exchange of H-ZSM-5 zeolite (Si/Al = 11.5) with AgNO_3_ solution. Following the ion exchange procedure, the Ag-zeolites were washed with either water or water and subsequently acetone, and then calcined (Ag_H2O_-ZSM-5 and Ag_Ac_-ZSM-5, respectively). The completeness of removal of all organic species from Ag_Ac_-ZSM-5 was confirmed^13^ (Figure S3). All zeolites, i.e., native H-ZSM-5, silver-loaded, then water or acetone washed, Ag_H2O_-ZSM-5 or Ag_Ac_-ZSM-5, resp., are found as high crystallinity zeolites (Figure S1; Table S1). There are no differences in their textural properties, which is consistent with the low silver content in Ag-zeolites. The presence of silver species has no negative effect on the micropore volume values, V_micro_, indicating that mass transfer of reactants within zeolite crystals remains unaffected. The first indicators of the differences in the degree of silver dispersion in Ag_H2O_- and Ag_Ac_-ZSM-5 are the changes in the color of the Ag-samples observed during vacuum-dehydration and subsequent hydration treatment (Method S1). The ion exchange process results in the substitution of H^+^ with silver cationic species, thereby affecting the picture of the hydroxyl groups (Figure S4). In both Ag-zeolites, the Si(OH)Al groups of the highest acidity are eliminated due to their replacement with silver cationic species. This is documented by the smaller half-widths of the Si(OH)Al groups bands and their location at higher wavenumbers, compared to H-ZSM-5. The narrowing of the Si(OH)Al groups band upon introducing silver cations and the high engagement of remaining OH groups in reaction with propylene can be deduced from available literature^11^; however, this has not been discussed. The Al-OH groups also anchor silver species to the zeolite surface, as indicated by a decrease in the Al-OH band (3660 cm^−1^). From a higher intensity of the Si(OH)Al group band in Ag_Ac_-ZSM-5, it can be anticipated that a smaller number of silver ions neutralise the negative charge of the zeolite framework, which consequently suggests the presence of silver sites of lower dispersion. Indeed, transmission electron microscopy studies confirmed the presence of encapsulated Ag nanoparticles of a bigger average size in Ag_Ac_-ZSM-5 (Figure S2). FT-IR spectroscopy employing probe molecules is well-suited for investigating surface sites. The selective investigation between probe and adsorption site is investigated, as the characteristics of the probe molecules can be accurately aligned with the properties and location of the surface active sites. Consequently, the information originates from sites accessible to reactants, particularly those involved in the catalytic process. In addition to Lewis sites originating from framework or extra-framework Al-atoms, the silver electron acceptor species also contribute to overall Lewis acidity; thus, the total acidity, both Brønsted (BAS) and Lewis (LAS), was probed with pyridine (Py) sorption. On the other hand, *in situ* FT-IR spectroscopy of CO adsorption was employed to get selective insight into the nature and dispersion of silver sites (Table S2). Py sorption data confirm the lower concentration of BAS in both Ag-zeolites. At the same time, a 4-fold and 2-fold increase in the number of Lewis acid sites is found for Ag_H2O_-ZSM-5 and Ag_Ac_-ZSM-5, respectively. The difference in LAS density in both Ag-zeolites indicates the diverse nuclearity of silver clusters in each zeolite. This is further confirmed by the CO sorption data, which prove the presence of Ag_n_^+^ clusters of higher nuclearity in Ag_Ac_-ZSM-5 (Table S2; Figure S4). The metallic phase is not detected, which is consistent with findings from XPS studies (Figure S5). The concentration of silver cations detected by carbon monoxide, Ag^+^(CO), was compared to the total number of silver atoms (ICP data), revealing that in Ag_H2O_-ZSM-5, the Ag_2_^+^ species are the most abundant. In contrast, in Ag_Ac_-ZSM-5, the framework is balanced by the Ag_3_^+^ species. The Ag_2_^+^ and Ag_3_^+^ species reflect the average nuclearity of silver clusters (Table S2).

**Figure 1:**
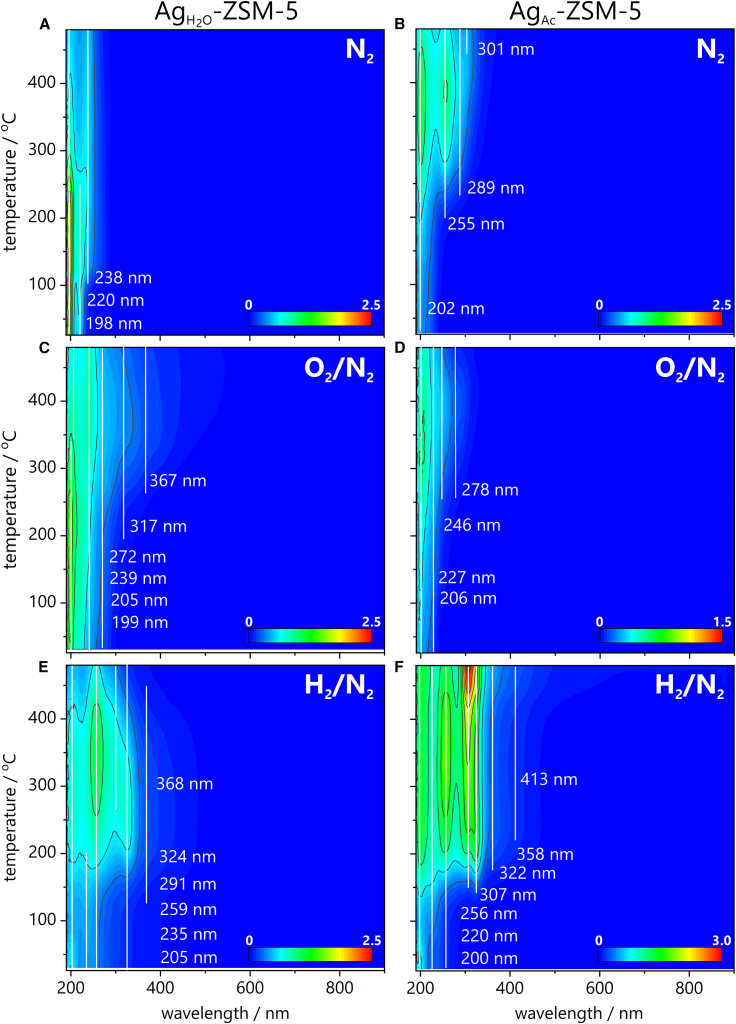
UV-vis spectra obtained during temperature-programmed treatment (A and B) under inert (N_2_), (C and D) under oxidizing (O_2_/N_2_), and (E and F) under reducing (H_2_/N_2_) atmosphere for Ag_H__2__O_-ZSM-5 (A, C, and E) and Ag_Ac_-ZSM-5 (B, D, and F). Scale bars: bands intensity in Kubelka-Munk [a.u.].

The speciation of silver in inert, oxidizing, and reducing atmospheres was investigated by *in situ* temperature-programmed UV-vis measurements. These studies were conducted in the presence of nitrogen (Figures 1A and 1B), synthetic air (Figures 1C and 1D), and hydrogen (10vol % in N_2_) (Figures 1E and 1F). The different dispersion of silver species resulting from using water or acetone during the washing step leads to the varying susceptibility of Ag-zeolites to oxidation and reduction. The Ag_3_^+^ sites in Ag_Ac_-ZSM-5 exhibit greater susceptibility to reduction and subsequent oxidation than the Ag_2_^+^ sites in Ag_H2O_-ZSM-5. In the case of Ag_Ac_-ZSM-5, unlike Ag_H2O_-ZSM-5, non-reversible formation of large nanoparticles is not observed.^14^ Interpreting UV-Vis spectra and accurately assigning the observed bands to specific silver species is challenging; however, the literature shows some agreement regarding these assignments. The bands below 240 nm are attributed to the Ag^+^ ions, while those above 350 nm are due to silver nanoparticles (Ag^0^ NPs) larger than 1 nm. The region in between cannot be precisely ascribed, only tentatively to the range of Ag_n_^δ+^ and Ag_m_ species. Usually, the bands at 250–270 nm are ascribed to Ag_8_^δ+^, while those around 310–325 nm are to Ag_8_^0^ clusters.^8^^,^^9^ The Ag_H2O_-ZSM-5 presented only minor changes upon heating in an inert (N_2_) atmosphere. The primary changes are observed for the bands at 198 nm and 220 nm, indicating the onset of aggregation above 100°C, and the final Ag_n>8_^δ+^ sites show the bands at 238 nm, seen at temperatures above 300°C. In Ag_Ac_-ZSM-5, Ag_n>8_^δ+^ are formed at lower temperatures; some undergo autoreduction at temperatures as high as 400°C, as confirmed by an upsurge of the Ag_n>8_^0^ band at 300 nm. The oxidizing atmosphere guarantees a high fraction of Ag^+^ for both samples. However, even in the presence of oxygen, Ag_H2O_-ZSM-5 undergoes slight autoreduction at temperatures above 400°C, resulting in Ag_n>8_^0^, as indicated by the band at 319 nm. The same species Ag_n>8_^0^ are produced through reduction with hydrogen at temperatures below 200°C. Compared to Ag_H2O_-ZSM-5, Ag_Ac_-ZSM-5 exhibits greater susceptibility to the oxidation and reduction of Ag species at temperatures below 400°C, without resulting in large nanoparticles.^8^

### The catalytic activity in the ethylene conversion process

**Figure 2:**
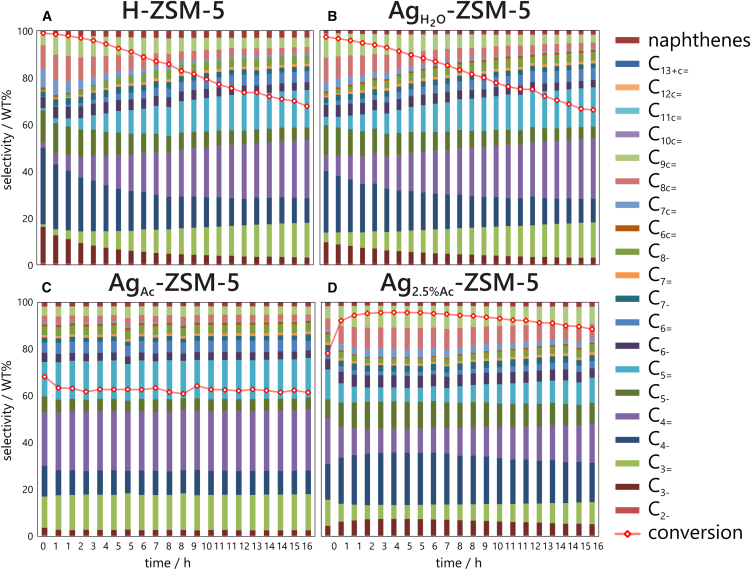
Ethylene conversion and selectivities (A) over H-ZSM-5, (B) over Ag_H__2__O_-ZSM-5, (C) over Ag_Ac_-ZSM-5, and (D) over Ag_2.5%Ac_-ZSM-5 samples at 350°C.

The activity of silver-loaded zeolites and reference H-zeolite was evaluated in the long-term catalytic tests at 350°C (Figure 2). The H-ZSM-5 shows a slow drop in conversion upon time on stream. In the high conversion range, the formation of paraffins and aromatics is favored, while at lower conversion, olefins are mainly formed. A notable difference (97% vs. 68%) in the initial ethene conversion is found between Ag_H2O_-ZSM-5 and Ag_Ac_-ZSM-5. The conversion on Ag_H2O_-ZSM-5 diminishes significantly over time, similar to H-ZSM-5. In contrast, over Ag_Ac_-ZSM-5, the ethylene conversion remains constant during a 16-h time-on-stream, yielding propylene, other olefins, and aromatics with approximately 85% selectivity. A steady conversion over 90% is also achieved with the zeolite with higher Ag content (2.5 wt.%, 230 μmol g^−1^), i.e., Ag_2.5%Ac_-ZSM-5. The selectivity for aromatics concurrently increases. Acetone-washed zeolites, i.e., Ag_Ac_-ZSM-5 and Ag_2.5%Ac_-ZSM-5, demonstrate markedly enhanced stability throughout the operational period compared to protonic or water-washed zeolites. The findings indicate that the acetone-assisted synthesis significantly and durably alters the silver distribution on the surface, thereby influencing catalyst performance.

The analysis of the quantitative results from temperature-programmed Py desorption FT-IR studies (Table S2), when examined alongside the activity and selectivity of the tested catalysts, suggests that the decrease in both the number and strength of Brønsted acid sites (Table S2) cannot be regarded as the sole factor affecting the catalytic parameters mentioned above. The difference in the quantity of Brønsted acid sites (BAS) for Ag_Ac_-ZSM-5 to H-ZSM-5 does not exceed 10%. A greater reduction in the concentration of Brønsted acid sites is observed for Ag_H2O_-ZSM-5, yet both Ag_H2O_-ZSM-5 and H-ZSM-5 demonstrate similar conversion and selectivity. Furthermore, Ag_H2O_-ZSM-5 and Ag_Ac_-ZSM-5 contain acid sites of nearly the same strength, showing no notable variation in the quantity of Brønsted acid sites, although their catalytic performance is dramatically different. Therefore, the strength of the Brønsted acid sites does not serve as a parameter that notably distinguishes activity. These considerations highlight the significant role of silver sites in ethylene oligomerization, especially regarding their nuclearity and localization.

### *Operando* UV-vis/FT-IR-GC-MS investigation of ethylene conversion over the studied catalysts

The spectroscopic insight into the interaction of reactants with the catalysts was combined with simultaneous GC and MS analysis of the products desorbing from the catalyst’s surface. This integrated approach enhances our understanding of the nature of the molecules formed on the catalyst surface and, consequently, the catalytic activity. UV-vis spectra collected for pre-oxidized Ag_Ac_-ZSM-5 sample before contact with ethylene demonstrate high heterogeneity of silver forms; isolated Ag^+^ cations, aggregated and nanosized species are distinguished (Figure S6A). In Ag_H2O_-ZSM-5, the predominant species are isolated Ag^+^ cations, whereas other forms are present in negligible amounts (Figure S6B).

The ethylene oligomerization reaction products, i.e., paraffins (m/z = 43), olefins (m/z = 41, 55, 56), and aromatics (m/z = 91), were monitored by mass spectroscopy (Figure S7). A delay in the formation of olefins and aromatics is observed for the H-ZSM-5 catalyst. Moreover, steady-state product formation is achieved in a reduced time for silver-loaded zeolites. In the initial reaction stage, the primary products for H-ZSM-5 are methane and propane. However for Ag-loaded zeolites, C_3_-C_4_ hydrocarbons, predominantly olefins, are formed, with propane being the most abundant (Figure S8). At later stages of ethylene oligomerization, the Ag_Ac_-ZSM-5 yields the most significant share of C_3_-C_4_ hydrocarbons, predominantly olefins; after 85 min of reaction, the share of C_5_-C_9_ increased further, and olefin production remained dominant. The protonic and Ag_H2O_-ZSM-5 zeolites in the later stages of the reaction produce a greater share of the heavier C_5_-C_9_ fraction and BTEX (benzene, toluene, ethylbenzene, xylenes) (Figure S8).

**Figure 3:**
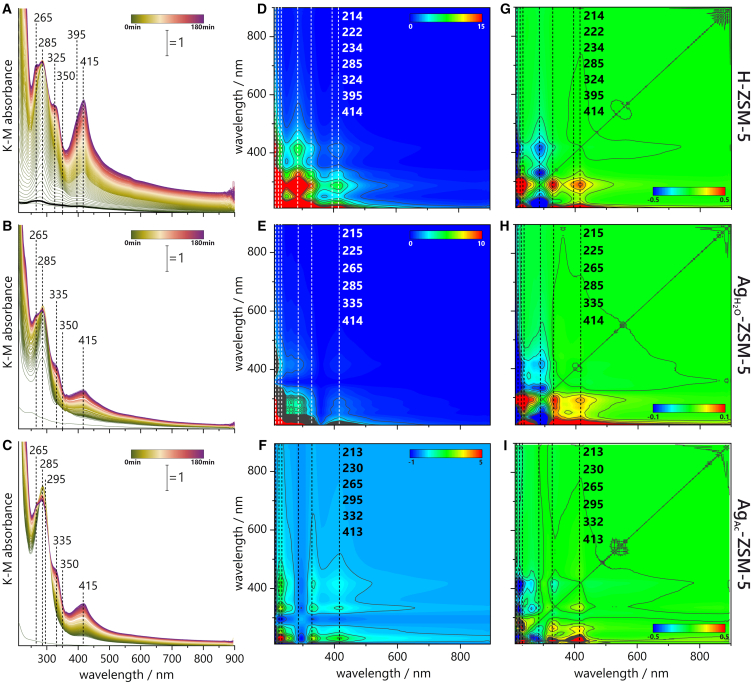
UV-vis spectra and 2D COS correlation maps of these spectra obtained during ethylene oligomerization (A–C) UV-Vis spectra, (D–F) synchronous maps, and (G–I) asynchronous maps. (A, D, and G) over H-ZSM-5, (B, E, and H) over Ag_H2O_-ZSM-5, and (C, F, and I) over Ag_Ac_-ZSM-5. Scale bars: (A–C) 180 min of reaction duration and Kubelka-Munk intensity [a.u.], (D) 15 a.u., (E) 10 a.u., (F) 6 a.u., (G) 1 a.u., (H) 0.2 a.u, and (I) 1 a.u. of correlation intensity.

The UV-vis spectra show many bands that emerge at particular reaction intervals depending on the catalyst employed (Figures 3A–3C). Regardless of the type of catalyst used (Ag-zeolites or H-ZSM-5), the primary UV-visible absorption bands appear at the same wavelengths. This suggests that Brønsted acid sites play a significant role in the transformation of ethylene. The oligomerization of alkenes into larger alkenes, isomerization, hydrogen transfer, and cracking (β-scission) are influenced by the density and strength of Brønsted acid sites, and reaction conditions such as temperature. However, the differences in growth of the intensities among the bands are attributed to the cooperative role of silver sites. The UV-vis signatures of hydrocarbons reported in the literature are often not unambiguous; therefore, a discussion on their assignment is presented (Table S3). The discussion in this article is based on the exquisite experiments of Wulfers and Jentoft^7^ who performed UV-vis characterization of intermediates, i.e., cyclopentenyl cations and others, formed during methanol conversion. Cyclopentenyl cations were also identified as active intermediates during propane aromatization on zeolite Ga/ZSM-5.^15^

In the initial period of ethylene oligomerization, the H-ZSM-5 exhibits the formation of neutral aromatics (<225 nm), such as benzene or toluene, and alkyl-substituted cyclopentenyl cations (285 nm). An induction period is recognized by a gradual increase of alkyl-substituted cyclopentenyl cations, while protonated acyclic alkyl-substituted trienes,^16^ highly alkylated benzenium ions or naphthalenic species,^17^ are observed only to a limited extent. The band at 395 nm, together with the band of alkylated naphthalenes (410 nm) or other large polycyclic aromatics (415 nm),^18^ is persistent against purging with inert gas, which suggests their confined sorption inside micropores (Figure S18). After a few minutes of reaction, the methylbenzenium ions (350 nm) and methylated naphthalene carbocations (395 nm) are produced. The last band gradually shifts to higher wavelengths, indicating the formation of polyalkylated naphthalenic species, i.e., coke precursors, over time. Unlike H-ZSM-5 and Ag_H2O_-ZSM-5, the Ag_Ac_-ZSM-5 catalyst does not show a discernible induction period. The formation of naphthalenic species is substantially inhibited. Moreover, the naphthalenic species reaches its maximum concentration at the onset of the reaction but exhibits a decrease over time. The most extensive formation of naphthalenic species is found in H-ZSM-5, i.e., the catalyst with the highest BAS concentration. The reduced ability of Ag-zeolites for naphthalenic species formation is attributed to (i) a reduced density of protonic sites and (ii) the presence of silver cations, which interact strongly with ethylene and subsequently with the cyclopentadiene intermediate, promoting alkene production over coke precursors.

A more precise understanding of the induction period in Ag-zeolites was gained through experiments with lower ethylene loading (Figure S9). It is important to highlight that the experimental conditions used in this study allow for the observation of the induction period involving alkyl-substituted cyclopentenyl cations (285 nm) and methyl/ethylbenzenium ions (350 nm). This time, similarly to H-ZSM-5, alkyl-substituted cyclopentenyl cations are the primary intermediates emerging during the induction period. The primary reaction products are methane and/or propane (Figure S8). Significantly, the rapid formation of cyclopentenyl cations and methyl/ethylbenzenium ions within Ag_Ac_-ZSM-5 helps to eliminate the induction period on H-ZSM-5.^7^ The alkyl-substituted cyclopentenyl cations are formed preferentially, thereby suppressing the formation of alkyl-substituted benzenium ions. The cyclopentenyl cations can originate from the re-protonation of dienes formed after removing four hydrogens from ethylene oligomers. This hydrogen abstraction process is driven by the transfer of hydrogen to alkene species within the zeolite, leading to a high yield of paraffins in the gaseous products.^19^ Once formed, the cyclopentenyl cations can interconvert to benzene or alkyl-substituted benzene through a rearrangement that removes four more hydrogens, generating further alkane products through hydrogen transfer to alkenes.^20^ The above-mentioned literature reports contradict the findings of this work, i.e., inhibited alkyl-substituted benzenium ion production during the synthesis of alkyl-substituted cyclopentenyl cations. Accordingly, 2D COS analysis is employed to gain insight into the surface chemistry during ethylene oligomerization (Figures 3D–3I; Figures S12–S15). The synchronous map derived from the UV-visible data allows for identifying events that occur simultaneously or coincide in time. The formation of alkyl-substituted benzenium ions (325 nm) is accompanied by further substitution of the alkyl group onto the benzene ring (395–400 nm)^7^ or the production of alkylated naphthalenic species (415 nm), as documented by positive cross-peaks between their bands.^21^^,^^22^ The negative correlations involving the band at 295 nm are exclusively observed for Ag_Ac_-ZSM-5. They point to the lack of stability of highly alkylated cyclopentenyl cations (295 nm) and their rapid conversion into neutral monoaromatic (265 nm) or naphthalenic (415 nm) species. Monoaromatic species undergo condensation reactions, finally yielding polycyclic aromatics. Neutral olefins and aromatics (235, 265 nm) appear before alkylated cyclopentenyl cations, as noted from the synchronous map. 2D COS results further reveal that alkyl-substituted benzenium ions (325 nm) and naphthalenes (395–400, 415 nm) bands are the primary species formed on the catalyst surface. This correlation is, however, very weak, and due to the extended accumulation time of the UV-vis spectrum, this observation requires validation through further studies.^21^^,^^22^

To improve the assessment of the intermediates of the ethylene oligomerization process on H- and Ag-ZSM-5 zeolites, *operando* UV-vis data were complemented by *operando* FT-IR spectroscopic investigations. FT-IR spectroscopy enables a more detailed understanding of surface phenomena on catalysts, offering higher time resolution compared to DR UV-Vis spectroscopy, as FT-IR spectra can be acquired within a few seconds. The attribution and comprehensive discussion of FT-IR bands are presented in Table S3. The consumption of the Si(OH)Al band is the first indicator of heavy product accumulation on the catalyst surface during the oligomerization of C_2_H_4_ (Figure S10). The Si(OH)Al groups in Ag_Ac_-ZSM-5 are more significantly affected by interaction with intermediate species than those in H-ZSM-5, even though the former has a lower concentration of protonic sites. However, these species cannot be categorized as coke species, as evidenced by the recovery of the Si(OH)Al group following product desorption. Thus, the moderate to low acidity of the Si(OH)Al sites, as indicated by the position of the Si(OH)Al band in Ag-zeolites, is thought to contribute to the enhanced stability of the Ag-zeolite catalysts.

**Figure 4:**
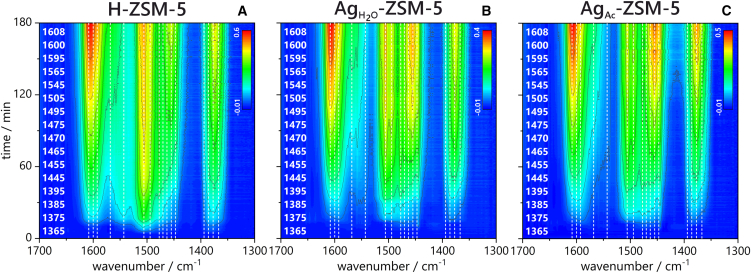
FT-IR spectra in the region 1650-1300 cm^−1^ obtained during ethylene oligomerization (A) over H-ZSM-5, (B) over Ag_H__2__O_-ZSM-5, and (C) over Ag_Ac_-ZSM-5. Scale bars: bands intensity [a.u.].

Additionally, the assignment of reactant bands in the C=C stretching and C-H deformation vibration regions (Figure 4), along with a detailed discussion, is presented in Table S3. Time-traces of specific IR bands are used to monitor their changes in the complex spectra (Figure S11). The FT-IR data clearly elucidate the surface chemistry of the H-ZSM-5 sample, showing that within the first few minutes, neutral conjugated alkenes^23^ and neutral polymethylbenzenes are formed.^24^ The appearance of aromatics is corroborated by detecting paraffins (methane, propane). The rapid formation of alkyl-substituted cyclopentenyl cation^7^^,^^25^ (the band of 4C=C at 1505 cm^−1^) is accompanied by a higher amount of olefins after the induction period (60–180 min). In the UV-vis spectra, the main spectroscopic feature of cyclopentenyl cations was observed at 285 nm.^6^^,^^26^^,^^27^ The relationship between UV-vis and FT-IR results excludes the 1505 cm^−1^ band from being associated with 1,2,4-trimethylbenzene, highlighting the reliability of the correlation between these methods in the studies of intermediate species. Our research also validated the attribution of the band at 1605 cm^−1^ as a reliable indicator of the presence of tetramethylbenzene ions, another key reactant in HP. What further distinguishes Ag-loaded zeolites from H-analogue is the pronounced emergence of CH_3_ δ_asym_(−CH_3_) and CH_2_ δ(−CH_2_) bands in 1475–1455 cm^−1^, which suggests increased production of compounds rich in CH_3_ and CH_2_ fragments, including iso-products and extensively alkylated aromatics. This is also confirmed by catalytic tests, where the isoC_4_/C_4=_ 3.6–4.8 was the highest for the Ag_Ac_-ZSM-5 sample (isoC_4_/C_4=_ 2.8–4.1 for H-ZSM-5 and 3.2–4.1 for Ag_H2O_-ZSM-5). Similar to the MTH reaction,^28^ the ring expansion of cyclopentenyl cations and subsequent dehydrogenation in ethylene oligomerization leads to aromatics. On the other hand, the carbenium ions formed as the intermediates in C_2_H_4_ oligomerization with the participation of the strong Brønsted acidic sites of zeolites have been found to isomerize to more energetically preferable secondary or tertiary ions, resulting in the formation of the branched oligomers. This considerably contradicts our IR findings, which reveal the presence of large amounts of iso-products in the Ag-zeolites. Therefore, the formation of the branched or highly alkyl-substituted intermediates is not ruled solely by the number and strength of protonic sites. This strongly highlights the role of Ag cations in ethylene binding and, ultimately, their influence on the selectivity of the ethylene oligomerization reaction. The [Ag(C_2_H_4_)^+^] and [Ag(C_3_H_6_)^+^] complexes are claimed to be stable up to 350°C, while at 550°C, the extensive formation of aromatic hydrocarbons is observed.^11^^,^^12^^,^^29^^,^^30^ Consequently, the formation of [Ag(C_2_H_4_)^+^] complexes appears to limit ethylene oligomerization and aromatization on Ag_Ac_-ZSM-5, whereas protonic zeolite H-ZSM-5 facilitates substantial naphthalene formation (415 nm). The [Ag(C_2_H_4_)^+^] π-complexes account for the selectivity of ethylene adsorption on Ag^+^ rather than on Brønsted acid sites in Ag-ZSM-5 samples.^31^ The formation of this complex results in two possible electronic ion contributions: π-donation from ethylene to Ag^+^ and π-back-donation from Ag^+^ d-electrons to the antibonding orbitals of ethylene. Thus, it is assumed that the synergistic effect between Brønsted acid sites and silver cations allows for the rapid formation of cyclopentenyl cations without the concurrent formation of polyaromatic compounds. This cooperative effect is more pronounced for Ag_Ac_-ZSM-5 zeolite with less dispersed Ag species, indicating the significant importance of the targeted nuclearity of Ag sites in ethylene binding. However, forming cyclopentadienyl anions over silver cations is an intriguing option for ethylene transformation over Ag-H zeolites.^32^

**Figure 5:**
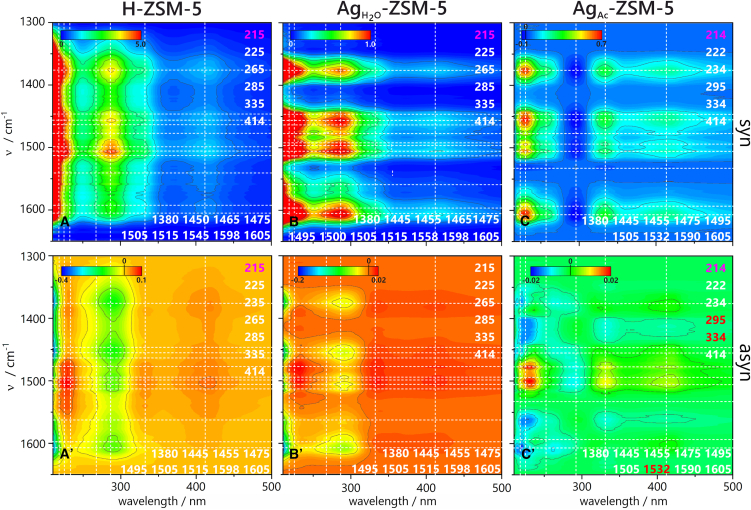
2D COS heterospectral correlation maps of FT-IR and UV-vis regions 1650–1300 cm^−1^ × 200–500 nm obtained during ethylene oligomerization (180 min) (A–C) synchronous maps, (A′–C′) asynchronous maps, (A and A') over H-ZSM-5, (B and B') over Ag_H2O_-ZSM-5, and (C and C') over Ag_Ac_-ZSM-5. Scale bars: a.u. of correlation intensities.

The IR data were further elucidated through 2D COS synchronous and asynchronous analysis (Figure 5). The identified correlations indicate that cyclopentenyl cations (the most intensive correlations for the band at 1505 cm^−1^) significantly influence the ethylene oligomerization process on H-ZSM-5 throughout all reaction stages. For Ag_H2O_- and Ag_Ac_-ZSM-5, the interaction of ethylene and other light olefins with silver species is equally important as the formation of cyclopentenyl cations. The accumulation of tetramethylbenzenium cations (ν_C=C_ band at 1605 cm^−1^ and δ_CH3_ and δ_CH2_ bands at 1385–1355 cm^−1^) in all catalysts precedes the formation of both cyclopentenyl cations and the complexes between silver and short olefins. The preferred accumulation of tetramethylbenzenium cations on the Ag-catalyst surface is also observed, which was previously confirmed by 2D COS analysis of *operando* UV-vis studies. Further, the formation of π-complexes between the Ag^+^ and olefins precedes the formation of cyclopentenyl cations. As a result, the ultimate products originate from the cyclopentenyl cations, the last intermediates seen on the catalyst surface. The accumulation and formation of neutral species become the predominant processes on the catalyst surface with longer reaction times, which is related to the slow deactivation of the catalysts. This observation again emphasizes the significant reactivity of cyclopentenyl cations during the HP process, which controls ethylene oligomerization. From 2D COS analysis, it can also be anticipated that the proton from the Si(OH)Al groups is being accepted by intermediate products created during the ethylene reaction. This effect cannot be attributed to coke deposit formation since there was no discernible decrease in activity throughout the catalytic tests or *operando* spectroscopic investigations. Therefore, active site poisoning and deactivation can be excluded as the processes consuming Brønsted acidity (Figure 2; Figure S8). Accordingly, the intensity of Si(OH)Al groups decreases after the formation of neutral polyenes and the [Ag(C_2_H_4_)^+^] complexes. The 2D COS maps do not allow for concluding the change order between the bands of the Si(OH)Al groups and cyclopentenyl cations.

### Heterospectral 2D COS analysis of FT-IR and UV-vis *operando* results of ethylene oligomerization

The 2D COS analysis can also be applied across spectroscopies of different wavelengths, herein the FT-IR and UV-vis. The heterospectral 2D COS analysis provides a solution to the fundamental problems associated with assigning IR and UV-Vis bands to the correct chemical species. To ensure the correct interpretation of data obtained from 2D COS analysis, the experimental procedures were identical, and the spectra were sampled to maintain a comparable timeline for their collection. The mid-IR spectra (1650–1300 and 3800–3500 cm^−1^) were correlated with the UV-vis spectra (200–500 nm) (Figure 5; Figure S15). This approach enables the identification of associated events by their corresponding IR and UV-vis bands. The results presented later in the discussion are the first reported attempt to apply the 2D COS analysis in heterospectral mode to heterogeneous catalytic studies.^33^

**Figure 6:**
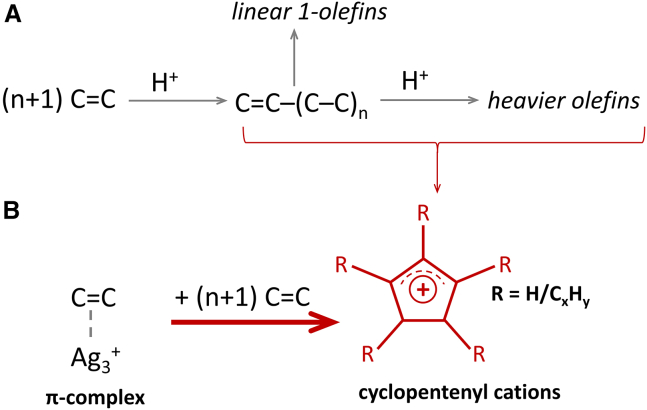
Schematic representation of the role of H^+^ and silver sites in cyclopentenyl cations during ethylene oligomerization (A) Homologation mechanism on H-ZSM-5.^34^ (B) Direct CP synthesis from π-complex on Ag_Ac_-ZSM-5.

For each sample, two synchronous and two asynchronous maps are presented to reveal the relationships between bands in the UV-vis and FT-IR spectral ranges (Figures S12–S14). The set of correlations found in 2D COS synchronous and asynchronous maps of 3800–3500 cm^−1^ x 200–500 nm regions (Figure S15) confirms that the accumulation of neutral olefins, aromatics (<235 nm, 265 nm), alkyl-substituted cyclopentenyl cations (285 nm), as well as alkyl-substituted benzenium ions and naphthalenes (330 and 415 nm), precedes the consumption of all types of O-H groups bands in the IR range. This effect is the most pronounced for the Ag_Ac_-ZSM-5 sample. The picture becomes more complicated when the 1650–1300 cm^−1^ and 200–500 nm regions are correlated (Figure 5). The robust correlation between the bands at 285 nm and 1505 cm^−1^ suggests that, for zeolite H-ZSM-5, these bands arise from the same species, i.e., cyclopentenyl ions. This further supports the attribution of the 1505 cm^−1^ band to cyclopentenyl cations. For Ag-zeolites, the correlation between IR and UV-vis bands proves that species represented by bands at ca. 1600 cm^−1^ assigned to neutral aromatics and dienes or trienes correlate strongly with all those found in the UV-vis region, with high contribution from alkyl-substituted benzenium ions (tetramethylbenzenium, 330 nm) and polyalkylnaphthalenes (415 nm). The evolution of all bands in the IR range occurs alongside the depletion of the 295 nm band in the UV-vis spectrum. Faster formation of cyclopentenyl cations corresponds to stronger correlations among bands representing other species in the spectrum. The ethylene oligomerization process thus serves as another example of the co-catalytic role of cyclopentenyl cations as active HP species, leading to the generation of aromatics. The high reactivity of cyclopentenyl cations in Ag_Ac_-ZSM-5 indicates that the reaction is primarily influenced by the HP reaction, which is governed by the presence of silver in particular dispersion. The formation of [Ag(C_2_H_4_)^+^] complexes limits ethylene oligomerization and aromatization over the Ag_Ac_-ZSM-5 sample, whereas on protonic zeolite extensive formation of naphthalenes, represented by the 415 nm band, was found. The cooperative effect of Ag_n_^+^ and H^+^ sites is more pronounced for Ag_Ac_-ZSM-5 zeolite with less dispersed Ag species, indicating the significant importance of the targeted nuclearity of Ag sites in ethylene binding. The role of Ag cations in ethylene binding (Figure 6), ultimately, and their influence on the selectivity of the ethylene oligomerization reaction is therefore emphasized.

### Insight into coke species formed over protonic and silver-loaded zeolites

The characterization of carbonaceous surface species is important due to their role as residues containing active sites for the alkylation or isomerization reactions. The coke species can also deteriorate the catalyst activity. The deactivation of catalysts in hydrocarbon transformation involves the formation of carbonaceous deposits that block the zeolite micropores (soft coke) or are located at the external surface of the zeolite (hard coke), thereby preventing access to the zeolite pores. The amount, type, and location of coke on the catalyst surface are fundamental properties that depend on various factors, including the number and location of active sites and the pore dimensions and topology. As evidenced by XPS studies of C1s of spent catalysts and GC-MS analysis of extracted coke (Figure S16), the carbonaceous species produced over protonic zeolite is more heterogeneous and contains more aliphatic, monoaromatic, and polyaromatic species than the coke species formed over Ag_Ac_-ZSM-5 spent catalyst. The coke of H-ZSM-5 zeolite contains fewer alkyl-substituted aromatic species compared to the coke of the Ag_Ac_-ZSM-5 sample.

**Figure 7:**
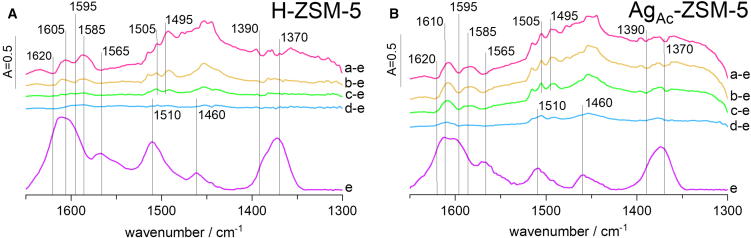
FT-IR spectra registered during 60 min of purging the catalysts after ethylene oligomerization (A) H-ZSM-5 catalyst. (B) Ag_Ac_-ZSM-5 catalyst. Spectra after 10, 20, 30, and 40 min of purging are presented as difference spectra (a-e, b-e, c-e, d-e). The FT-IR spectrum (e), recorded after 60 min, is presented in its direct form. As a result, the different spectra represent the species removed from the catalyst’s surface in successive steps after 10 min, then between 10 and 20, 20 and 30, and 40. The direct spectrum represents species not removed upon purging with helium for 60 min. Scale bars: bands intensity [a.u.].

The coke was also assessed for the samples after *operando* FT-IR-GC-MS spectroscopic investigations. In the FT-IR spectra collected during the purging of the catalyst surface with helium (Figure 7), a decrease in bands representative of -CH_3_ (1390, 1370 cm^−1^), -CH_2_ (1460–1440 cm^−1^), and aromatic species (1495 cm^−1^) is observed. The band of cyclopentenyl cations (1505 cm^−1^) also decreases during purging with helium, suggesting that those species are unstable during the reaction and easily transform into either neutral monoaromatic or polyaromatic species (PAS). Similar conclusions were drawn from UV-vis spectra recorded during purging conditions (Figure S18). Conversely, the IR bands characteristic for naphthalenes (1585 and 1565 cm^−1^) exhibit a slight rise, indicating the formation of polycyclic aromatic compounds from olefins and monoaromatic species of hydrocarbon pool components. The most significant formation of PAS is found in the protonic H-ZSM-5 sample.

Following this, the coke oxidation process is investigated through FT-IR-TPO experiments, with mass spectrometry employed to analyze CO_2_, CO, and H_2_O in the outlet gases (Figure S17). In conclusion, silver markedly changes the nature of coke species by limiting the formation of mono- and polyaromatic compounds while promoting the development of strongly alkyl-substituted aromatics, which may serve as precursors for olefin production through the hydrocarbon pool mechanism. Furthermore, coke generation is restricted in silver-loaded samples, and the oxidation of coke species is enhanced. Consequently, the Ag_Ac_-ZSM-5 catalyst demonstrates distinctive stability during ethylene oligomerization, as observed in catalytic studies.

### Conclusions

The presented results covered three distinct topics: firstly, related to silver and its speciation, unexpectedly but strongly modified by acetone wash; secondly, the role of silver species in ethylene transformation at a temperature identified as transitional for metal-olefin complex stability over silver cations; and lastly, the similarities between the spectroscopic signatures of ethylene transformation intermediate species and hydrocarbon pool species. Comprehensive *in situ* FT-IR and UV-vis spectroscopic characterization, supported by 2D COS analysis, provided strong evidence of altered silver species dispersion after acetone washing, with Ag_3_^+^ predominating in the Ag_Ac_-ZSM-5 sample. This resulted in highly stable activity and selectivity during ethylene conversion at 350°C for 16 h. The different catalytic activity was followed by the various nature of coke in protonic and silver catalysts; the spent Ag_Ac_-ZSM-5 catalyst was rich in highly alkyl-substituted aromatics, with overall lower coke content than protonic H-ZSM-5. The FT-IR and UV-vis *operando* spectroscopic studies of ethylene conversion over catalysts provided insight into the nature of species formed. For the first time, 2D COS analysis in heterospectral mode, involving the simultaneous examination of two distinct spectral ranges, was applied to *operando* spectroscopy results in heterogeneous catalysis studies. This approach helped validate the spectroscopic signatures of cyclopentenyl cations, specifically the 285 nm band in UV-vis and the 1505 cm^−1^ band in FT-IR spectroscopy. The cyclopentenyl cations are critical intermediates in ethylene transformation, much like the hydrocarbon pool mechanism during methanol conversion.

### Limitations of the study

This study investigates the changes in silver speciation within ZSM-5 zeolite that occur when acetone is used during the ion exchange procedure. Zeolites of the same structure, with identical aluminum concentration and organization in the framework, and the same silver loading were examined, differing solely in the washing procedure, which used water and acetone interchangeably. The efficiency of the ion exchange process is also influenced by the arrangement and organization of aluminum atoms, which is determined by the Si/Al ratio and the zeolite synthesis protocol. Consequently, the final distribution of silver species will depend on these factors. Furthermore, the topology of zeolites influences the distribution of silver ions. Therefore, the impact of acetone treatment can be evaluated concerning zeolites of identical composition and structure, which limits the applicability of this study’s findings to other zeolites to some extent.

Another potential limitation of this study is its reliance on the quality of the collected spectroscopic data. It is essential to ensure a high signal-to-noise ratio and sufficiently high spectral resolution. Additionally, only spectra acquired under identical conditions are suitable for 2D COS analysis. The type and location of the active sites of the catalyst must be the only factor influencing the course of the catalytic reaction. This work emphasized the importance of these specific conditions that limit the applicability of the 2D COS approach. Many publications often misuse this approach because they use spectra obtained at different temperatures.

## Resource availability

### Lead contact

Further information and requests for resources and reagents should be directed to and will be fulfilled by the lead contact, Kinga-Góra-Marek (kinga.gora-marek@uj.edu.pl).

### Materials availability

All reagents in this study are commercially available. All materials can be prepared as described in the STAR Methods section.

### Data and code availability

• Data concerning structural, textural, acidic-redox properties and *operando* catalytic data have been deposited at https://doi.org/10.57903/UJ/QJ3KFP (https://uj.rodbuk.pl). Accession numbers are listed in the key resources table.
• This article does not report original code.
• Any additional information required to reanalyse the data reported in this article is available from the lead contact upon request.

## Acknowledgments

This work was funded by the “Young Labs” grant under the program “Excellence Initiative—Research University” at Jagiellonian University. Coke species analysis in zeolites was financed by Grant No. 2020/37/B/ST4/01215 from the National Science Centre, Poland. 2D COS UV-VIS-IR analysis was financed by Grant No, 2021/41/B/ST4/00048 from the National Science Centre, Poland. Editorial and writing help from dr Agata Olszewska is gratefully appreciated.

## Author contributions

Conceptualization K.G.M., K.T., F.R., and J.M.T.; data curation K.T., A.K., M.S.U., O.R., and A.W.; formal analysis K.T., A.K., G.J.L., M.S.U., O.R., and A.W.; funding acquisition K.G.M. and K.T.; investigation K.T., A.K., G.J.L., M.S.U., O.R., and A.W.; methodology K.G.M. and K.T.; project administration K.G.M. and K.T.; supervision K.G.M. and K.T.; visualization G.J.L., A.K., and K.T.; writing – original draft K.G.M. and K.T.; writing – review and editing K.T., K.G.M., O.R., and A.W.

## Declaration of interests

The authors declare no competing interests.

## Declaration of generative AI and AI-assisted technologies in the writing process

During the preparation of this work, the authors used Grammarly to ensure proper grammar and language correctness. After using this tool, the authors reviewed and edited the content as needed and take full responsibility for the content of the publication.

## STAR★Methods

### Key resources table

**Table:** 

REAGENT or RESOURCE	SOURCE	IDENTIFIER
**Chemicals, peptides, and recombinant proteins**		

NH_4_ZSM-5	Zeolyst	CBV2314
AgNO_3_	Sigma-Aldrich	CAS: 7761-88-8
Acetone	Sigma-Aldrich	CAS: 67-64-1
HCl	Sigma-Aldrich	CAS: 7647-01-0
HF	Honeywell Fluka	CAS: 7664-39-3
BaSO_4_	Nacalai Tesque	CAS: 7727-43-7
Pyridine	Sigma-Aldrich	CAS: 110-86-1
CO, 99.95%	Linde Gas	CAS: 630-08-0
SiC	Sigma-Aldrich	CAS: 409-21-2
N_2_	SIAD	CAS: 7727-37-9
C_2_H_4_	Praxair	CAS: 74-85-1
H_3_BO_3_	Sigma-Aldrich	CAS: 10043-35-3
CH_2_Cl_2_	Supelco	CAS: 75-09-2
O_2_/He 10 %, mixture	Linde Gas	N/A

**Deposited data**		

Structure, textural, and acidity factors of H- and Ag-ZSM-5 zeolites applied for ethylene conversion. The detailed spectroscopic insight from operando FT-IR-UV-vis spectroscopy and 2D COS analysis.	This paper	UJ RODBUK: https://doi.org/10.57903/UJ/QJ3KFP (https://uj.rodbuk.pl)

### Experimental model and study participant details

This study does not use experimental models.

### Method details

#### Catalyst preparation

Catalyst NH_4_ZSM-5 (Zeolyst CBV2314, Si/Al = 11.5) was obtained commercially. The sample was calcined at a rate of 2°C·min^−1^ to 350°C (kept for 3 h), and then the temperature was increased at a rate of 1.5°C·min^−1^ to 550°C (kept for 4 h). The Ag-exchange procedure was performed solely on a protonic form of ZSM-5. For that, AgNO_3_ of the proper amount for 2 wt% Ag content was dissolved in 100 ml of deionised water, and 1 g of zeolite was added and stirred at RT for 96 h without light exposure. After the ion exchange procedure, the zeolites were filtered on a Büchner funnel and washed with 50 ml of either water (Ag_H2O_-ZSM-5-nc) or water and then acetone (Ag_Ac_-ZSM-5-nc). Next, samples were dried at 100°C for 24 hours and calcined at 550°C for 2 h (Ag_H2O_-ZSM-5 and Ag_Ac_-ZSM-5). This procedure was sufficient to remove all organic species from Ag_Ac_-ZSM-5.

#### Catalyst characterization

The powder X-ray diffraction (XRD) patterns were measured on a Rigaku Multiflex diffractometer (Cu Kα, 40 kV, 40 mA). For crystallinity assessment, the sum of the integral intensity of the most intense reflections within the 2Θ angle range from 21.5° to 25.5°, to the sum of the integral intensity for the protonic form, calcined CBV2314, was calculated.

The low-temperature nitrogen sorption measurements were performed on a Quantachrome Autosorb–1 -MP gas sorption analyser. The pre-treatment of samples involved heating to 350°C and evacuation under high-vacuum conditions (10-5 mbar) for 16 hours. The micropore volume (V_micro_) was calculated using the *t*-plot method. The specific surface area (S_BET_) was calculated based on the Brunauer–Emmet–Teller (BET) method, following the Rouquerol et al. recommendations.^15^ The pore size distribution was calculated from the adsorption isotherm branch using the Barrett–Joyner–Halenda (BJH) model.

The Si, Al, and Ag content were measured by Inductively Coupled Plasma–Optical Emission Spectroscopy (ICP-OES, PerkinElmer, Optima 2100DV). Typically, 50-80 mg of sample was digested in a Teflon vessel using HCl (35%) and HF (48%) in a 5:2 ratio. The final concentration was adjusted with deionised water.

The X-ray photoelectron measurements were performed in ultrahigh vacuum (UHV). All the samples were evacuated in the pre-treatment chamber at a pressure lower than 10^-6^ mbar, heating slowly from room temperature to 300°C, and stabilised for 1 hour. The X-ray photoelectron spectra (XPS) were measured on a Prevac photoelectron spectrometer equipped with a hemispherical VG SCIENTA R3000 analyser. The photoelectron spectra were measured using a monochromatic aluminium Al Kα source (E = 1486.6 eV) and a low-energy electron flood gun (FS40APS) to compensate for the charge on the surface of the non-conductive samples. The base pressure in the analysis chamber during the measurements was 5 · 10^-9^ mbar. The spectra were recorded with a constant pass energy of 100 eV for the survey and for the high-resolution spectra. The binding energy scale was referenced to clean gold’s Au 4f 7/2 peak (84.0 eV). The binding energies (BE) of the core levels O 1s, Si 2p, C 1s (for spent catalysts), and Ag 3d were measured. The Si 2p peak at 103.0 eV binding energy was taken as an internal reference. Using X-ray photoelectron spectroscopy (XPS), the chemical and electronic states of a sample’s elemental components are analysed at a penetration depth of approximately 1 to 10 nm. Due to the aforementioned limitations and low Ag-loading in our samples, the Auger signals exhibited very low intensities; hence, the modified Auger parameters cannot be calculated reliably.

Silver status in the samples was established in UV-vis *in situ* spectroscopic investigation. The measurements were performed using a Shimadzu UV-2600 spectrometer, recording spectra in the 190-900 nm wavelength range. Self-supported pellets with masses of approximately 10 mg of pure zeolites were placed in a Praying Mantis® DRIFT accessory, equipped with a high-temperature reaction chamber (Harrick Scientific, USA) with KBr windows. Before measurement, the background was measured using a BaSO_4_ self-supported pellet as a reference sample. The reactor was heated to 500°C, with a ramp rate of 6°C·min^-1^ in a flow (30 ml·min^-1^) of nitrogen, synthetic air, or hydrogen (10%vol in He).

A high-resolution FEI Tecnai Osiris analytical transmission electron microscope, equipped with an X-FEG Schottky field emitter (operating at an accelerating voltage of 200 keV), was used for nanoscale imaging. Z-contrast images were acquired using a high-angle annular dark field (HAADF) detector in scanning transmission electron microscopy (STEM) mode. To maximise HAADF signal intensity, a spot size of approximately 1.5 nm and a camera length of 43 mm were used. High-resolution TEM images were recorded using a Rio CMOS camera (4 K × 4 K). Prior to microscopic analysis, the samples were dispersed on a lacey carbon film supported on a gold grid (Agar Scientific, 400 mesh)

Acid-redox sites were characterised by FT-IR *in situ* spectroscopy. All the IR spectra presented in this work were measured in transmission mode and normalised to an identical sample mass (10 mg). The acidic and redox properties of the protonic and silver-modified samples were investigated througth quantitative FT-IR experiments of pyridine (Py) and carbon monoxide sorption to probe the Brønsted and Lewis acid sites and the silver sites, respectively. The samples of pure zeolites, without adding any other compounds, were pressed into self-supported discs and then placed in a custom-made thermoregulated quartz IR cell. *In situ* evacuation at 500°C under high vacuum (10^-5^ mbar) for 1 h was performed to remove any physisorbed species. The probe molecules were introduced after cooling to the temperature required for Py or CO sorption experiments. To probe all acid sites, the sorption of Py excess was realised at 170°C under static conditions. Next, the gaseous and physisorbed molecules were removed by desorption at 170°C. These spectra were used for quantitative assessment of Brønsted and Lewis acid sites concentrations with the use of respective absorption coefficients (bands at 1545 cm^-1^ (pyridinium ions, PyH^+^) - 0.07 cm^2^·μmol^-1^; 1455-1442 cm^-1^ (Py coordinatively bonded to Lewis sites, PyL) - 0.10 cm^2^·μmol^-1^).^16^ The acid strength of Brønsted acid sites was assessed in Py thermal desorption experiments, in which a sample was heated to 330°C, maintained at that temperature for 15 min, and subsequently cooled to 170°C to capture the IR spectra. The ratio between the intensities of the Py bands at 330 170^◦^C and 170^◦^C indicates the relative strength of the Brønsted acid sites.

The sorption of carbon monoxide (CO, Linde Gas Poland, 99.95%) at room temperature allowed for interaction of CO exclusively with silver sites, which allowed for distinguishing between the oxo-species and cation-exchanged silver species.^35^ All spectra presented in this work were recorded by gathering 100 scans with a spectral resolution of 2 cm^-1^ on a Vertex 70 spectrometer (Bruker) equipped with an MCT detector. Based on the methodology of quantitative measurement developed for silver sites in zeolites,^35^ the concentrations of isolated silver ions were determined.

#### Catalytic tests

For FT-IR & UV-vis *operando* studies, the calcined H-ZSM-5 and its Ag-loaded counterparts were formed into self-supported pellets (25 mg) and placed in a spectroscopic cell. For the UV-vis experiment, the Praying Mantis™ cell was used. For FT-IR measurements, the HT-IRS 01 cell was applied (MeasLine, www.measline.com, Patent no. PL 232633). Gases were introduced to spectroscopic cells by 1/16” Teflon lines, kept at 100°C. The catalysts were heated under a flow of N_2_ (24 ml٠min^-1^) to 500°C (rate of 2°C٠min^-1^), and this temperature was kept for 45 min. Then, the cell was cooled to 350°C, and ethylene (2 ml٠min^-1^) and nitrogen (22 ml٠min^-1^) were introduced to a spectroscopic cell. The reaction was carried out for 180 min. The operando studies were realised using either Shimadzu UV-2600 or Vertex 70 Bruker FT-IR spectrometer. Gas chromatography (Agilent Technologies 7890B) and mass spectrometry (MeasLine, www.measline.com, RGA200) were used to assess the reaction products. The outlet gas analysis was carried out with mass spectrometry to identify the presence of short paraffins (m/z = 43), short olefins (m/z = 41, 55, 56), and aromatics (m/z = 91). After the catalytic reaction, the spectroscopic cell was purged with N_2_ for 30 min or cooled to room temperature.

For the catalytic test of ethylene conversion, the catalyst was pelletized, crushed, and sieved into 0.2–0.4 mm particle size. The 120 mg of sample was mixed with 1 g SiC (Sigma-Aldrich, 0.6–0.8 mm) before being introduced into a fixed-bed reactor. The catalyst was first activated with a nitrogen flow of 80 ml٠min^-1^ for 1 h at 550°C, and then the temperature was decreased to the reaction condition (350°C), and gases were switched to nitrogen (24 ml٠min^-1^). After temperature stabilisation, the flow of nitrogen (16 ml٠min^-1^) and C_2_H_4_ (8 ml٠min^-1^) was settled. Each experiment was analysed using an online gas chromatograph (BRUKER 450GC, with PONA plot capillary columns and FID detector) at the reactor outlet. Conversion and selectivity were considered on a carbon basis, with a carbon balance higher than 95% for each experiment.

#### 2D COS analysis of spectroscopic results

Due to the complexity of ethylene oligomerization, a two-dimensional correlation analysis applied to UV-vis (2D COS UV-vis) and FT-IR spectroscopic data (2D COS IR) is helpful in monitoring the formation of intermediates on a catalytically active surface. 2D maps with contour plots along two axes (wavenumbers and correlation intensity) enable better monitoring of the reaction as a function of time on stream than conventional 1D spectra with numerous overlapping bands. The auto-peaks found on the diagonal of 2D synchronous maps (2D sync-map) indicate the level of changes at a given band location. Furthermore, the direction of band intensity change (at *λ*_1_ or ν_1_ and *λ*_2_ or ν_2_) and the sequential order of these changes can be gained only from the tandem analysis of 2D synchronous and asynchronous maps (2D async-map). On synchronous maps, the positive correlation of cross-peaks located at a given position indicates that the intensity changes of two bands at the position are occurring in the same direction. In contrast, the negative sign of the cross-peak indicates that one of the bands increases its intensity at the expense of the other. Asynchronous maps with cross-peaks at the positions matching those identified on synchronous maps provide information on the order of changes observed for these bands. Again, the sign of cross-peaks is important for establishing the order of events. The identical sign of cross-peaks on both 2D sync- and async-maps informs that the band located at *λ*_1_ (ν_1_), lower wavelength, changes its intensity before the one located at λ_2_ (ν_2_), higher wavelength. If the sign of matching cross-peaks on 2D synchronous and asynchronous maps is opposite, then the order of changes is also opposite to the one described above.

#### Coke analysis

The following procedure was applied to analyse the coke species adsorbed on the catalyst surface. Spent catalysts were ground in an agate mortar and subjected to GC-MS and IR-TPO analysis. For GC-MS analysis, the zeolite (approximately 16 mg) was placed in a Teflon container and mixed with hydrofluoric acid (HF, 40% Honeywell Fluka, 1 ml). Then, the mixture was left for 1 hour. After the zeolite dissolution, H_3_BO_3_ (6 ml of 20% boric acid) was added, and after another hour, coke extraction was conducted using 1 g of dichloromethane (CH_2_Cl_2_). The chromatograms with mass spectroscopy results were analysed using the NIST database for extracted species identification.

To assess the nature of the carbon deposit after FT-IR spectroscopic studies, the 10 %O_2_/He gas mixture was introduced (30 ml٠min^-1^), and the temperature was gradually increased to 300, 350, 400, 450, 500, and 550°C with a rate of 10°C٠min^-1^. At each step the temperature was stabilised for 15 min. The outlet gas composition was followed by mass spectrometry (*m*/*z* = 18 (water), 28 (carbon monoxide), and 44 (carbon dioxide)).

The XPS measurements were performed on selected spent catalysts to assess the difference in C 1s signals of coke species.

### Quantification and statistical analysis

This study does not include statistical analysis or quantification.

### Additional resources

This study has not generated or contributed to a new website/forum and it is not part of a clinical trial.

## References

[1] Dai W., Sun X., Tang B., Wu G., Li L., Guan N., Hunger M. (2014). Verifying the mechanism of the ethene-to-propene conversion on zeolite H-SSZ-13. J. Catal..

[2] Lee K., Cha S.H., Hong S.B. (2016). MSE-Type Zeolites: A Promising Catalyst for the Conversion of Ethene to Propene. ACS Catal..

[3] Blay V., Epelde E., Rubén M., Perea L.A. (2018). Converting olefins to propene: Ethene to propene and olefin cracking. Catal. Rev..

[4] Koyama T.-r., Hayashi Y., Horie H., Kawauchi S., Matsumoto A., Iwase Y., Sakamoto Y., Miyaji A., Motokura K., Baba T. (2010). Key role of the pore volume of zeolite for selective production of propylene from olefins. Phys. Chem. Chem. Phys..

[5] Epelde E., Ibañez M., Aguayo A.T., Gayubo A.G., Bilbao J., Castaño P. (2014). Differences among the deactivation pathway of HZSM-5 zeolite and SAPO-34 in the transformation of ethylene or 1-butene to propylene. Microporous Mesoporous Mater..

[6] Hernandez E.D., Jentoft F.C. (2020). Spectroscopic Signatures Reveal Cyclopentenyl Cation Contributions in Methanol-to-Olefins Catalysis. ACS Catal..

[7] Wulfers M.J., Jentoft F.C. (2014). The Role of Cyclopentadienium Ions in Methanol-to-Hydrocarbons Chemistry. ACS Catal..

[8] Collett C.H., McGregor J. (2016). Things go better with coke: the beneficial role of carbonaceous deposits in heterogeneous catalysis. Catal. Sci. Technol..

[9] Lee K., Hong S.B. (2019). Hydrocarbon Pool Mechanism of the Zeolite-Catalyzed Conversion of Ethene to Propene. ACS Catal..

[10] Yang H., Ma C., Zhang X., Li Y., Cheng J., Hao Z. (2018). Understanding the Active Sites of Ag/Zeolites and Deactivation Mechanism of Ethylene Catalytic Oxidation at Room Temperature. ACS Catal..

[11] Lashchinskaya Z.N., Gabrienko A.A., Prosvirin I.P., Toktarev A.V., Stepanov A.G. (2023). Effect of Silver Cations on Propene Aromatization on H-ZSM-5 Zeolite Investigated by 13 C MAS NMR and FTIR Spectroscopy. ACS Catal..

[12] Hsieh M.-F., Zhou Y., Thirumalai H., Grabow L.C., Rimer J.D. (2017). Silver-Promoted Dehydroaromatization of Ethylene over ZSM-5 Catalysts. ChemCatChem.

[13] Kubelková L., Nováková J. (1991). Temperature-programmed desorption and conversion of acetone and diethyl ketone preadsorbed on HZSM-5. Zeolites.

[14] Hawkins A.P., Zachariou A., Parker S.F., Collier P., Howe R.F., Lennon D. (2021). Studies of propene conversion over H-ZSM-5 demonstrate the importance of propene as an intermediate in methanol-to-hydrocarbons chemistry. Catal. Sci. Technol..

[15] Rouquerol J., Avnir D., Fairbridge C.W., Everett D.H., Haynes J.M., Pernicone N., Ramsay J.D.F., Sing K.S.W., Unger K.K. (1994). Recommendations for the characterization of porous solids (Technical Report). Pure Appl. Chem..

[16] Sadowska K., Góra-Marek K., Datka J. (2012). Hierarchic zeolites studied by IR spectroscopy: Acid properties of zeolite ZSM-5 desilicated with NaOH and NaOH/tetrabutylamine hydroxide. Vib. Spectrosc..

[17] Tarach K., Góra-Marek K., Chrzan M., Walas S. (2014). Quantification of Silver Sites in Zeolites: Carbon Monoxide Sorption Monitored by IR Spectroscopy. J. Phys. Chem. C.

[18] Seifert R., Kunzmann A., Calzaferri G. (1998). The Yellow Color of Silver-Containing Zeolite A. Angew. Chem., Int. Ed. Engl..

[19] Dai W., Wu G., Li L., Guan N., Hunger M. (2013). Mechanisms of the Deactivation of SAPO-34 Materials with Different Crystal Sizes Applied as MTO Catalysts. ACS Catal..

[20] Olsbye U., Svelle S., Bjørgen M., Beato P., Janssens T.V.W., Joensen F., Bordiga S., Lillerud K.P. (2012). Conversion of Methanol to Hydrocarbons: How Zeolite Cavity and Pore Size Controls Product Selectivity. Angew. Chem., Int. Ed..

[21] Qian Q., Vogt C., Mokhtar M., Asiri A.M., Al-Thabaiti S.A., Basahel S.N., Ruiz-Martínez J., Weckhuysen B.M. (2014). Combined Operando UV/Vis/IR Spectroscopy Reveals the Role of Methoxy and Aromatic Species during the Methanol-to-Olefins Reaction over H-SAPO-34. ChemCatChem.

[22] Goetze J., Meirer F., Yarulina I., Gascon J., Kapteijn F., Ruiz-Martínez J., Weckhuysen B.M. (2017). Insights into the Activity and Deactivation of the Methanol-to-Olefins Process over Different Small-Pore Zeolites As Studied with Operando UV–vis Spectroscopy. ACS Catal..

[23] Ganjkhanlou Y., Berlier G., Groppo E., Borfecchia E., Bordiga S. (2017). In Situ Investigation of the Deactivation Mechanism in Ni-ZSM5 During Ethylene Oligomerization. Top. Catal..

[24] Gołąbek K., Tarach K.A., Góra-Marek K. (2017). Standard and rapid scan infrared spectroscopic studies of o-xylene transformations in terms of pore arrangement of 10-ring zeolites – 2D COS analysis. Dalton Trans..

[25] Stepanov A.G., Sidelnikov V.N., Zamaraev K.I. (1996). In Situ 13C Solid-State NMR and Ex Situ GC–MS Analysis of the Products of tert-Butyl Alcohol Dehydration on H-ZSM-5 Zeolite Catalyst. Chem.—Eur. J..

[26] Hernandez E.D., Manookian B., Auerbach S.M., Jentoft F.C. (2021). Shape-Selective Synthesis of Alkylcyclopentenyl Cations in Zeolites and Spectroscopic Distinction of Constitutional Isomers. ACS Catal..

[27] Manookian B., Hernandez E.D., Baer M.D., Mundy C.J., Jentoft F.C., Auerbach S.M. (2020). Experimental and DFT Calculated IR Spectra of Guests in Zeolites: Acyclic Olefins and Host–Guest Interactions. J. Phys. Chem. C.

[28] McCann D.M., Lesthaeghe D., Kletnieks P.W., Guenther D.R., Hayman M.J., Van Speybroeck V., Waroquier M., Haw J.F. (2008). A Complete Catalytic Cycle for Supramolecular Methanol-to-Olefins Conversion by Linking Theory with Experiment. Angew. Chem., Int. Ed. Engl..

[29] Uslamin E.A., Saito H., Kosinov N., Pidko E., Sekine Y., Hensen E.J.M. (2020). Aromatization of ethylene over zeolite-based catalysts. Catal. Sci. Technol..

[30] Gabrienko A.A., Arzumanov S.S., Moroz I.B., Toktarev A.V., Wang W., Stepanov A.G. (2013). Methane Activation and Transformation on Ag/H-ZSM-5 Zeolite Studied with Solid-State NMR. J. Phys. Chem. C.

[31] Kolokolov D.I., Arzumanov S.S., Freude D., Haase J., Stepanov A.G. (2016). Mobility of Stable π-Complexes of Ethylene with Ag+ Cations in Ag/H-ZSM-5 Zeolite: A 2H Solid-State NMR Study. J. Phys. Chem. C.

[32] Bencze É., Lokshin B.V., Mink J., Herrmann W.A., Kühn F.E. (2001). Vibrational spectra and structure of the cyclopentadienyl-anion (Cp−), the pentamethylcyclopentadienyl-anion (Cp∗−) and of alkali metal cyclopentadienyls CpM and Cp∗M (M=Li, Na, K). J. Organomet. Chem..

[33] Park Y., Jin S., Noda I., Jung Y.M. (2023). Continuing progress in the field of two-dimensional correlation spectroscopy (2D-COS), part II. Recent noteworthy developments. Spectrochim. Acta Mol. Biomol. Spectrosc..

[34] Chowdhury A.D., Lucini Paioni A., Whiting G.T., Fu D., Baldus M., Weckhuysen B.M. (2019). Unraveling the Homologation Reaction Sequence of the Zeolite-Catalyzed Ethanol-to-Hydrocarbons Process. Angew. Chem., Int. Ed. Engl..

[35] Tarach K., Gora-Marek K., Chrzan M., Walas S. (2014). Quantification of Silver Sites in Zeolites: Carbon Monoxide Sorption Monitored by IR Spectroscopy. J. Phys. Chem. C.

