# Supplementary material for: Unrevealed effect of silver species on hydrocarbon pool in ethylene conversion on ZSM-5 by 2D COS FT-IR and 2D COS UV-vis operando spectroscopies
Source: iScience. 2025 Sep 25;28(11):113650. doi: 10.1016/j.isci.2025.113650 (PMC12549377; doi:10.1016/j.isci.2025.113650)
Supplement: Document S1. Figures S1–S18 and Tables S1–S3 [file mmc1.pdf]

iScience, Volume *28*

Supplemental information

Unrevealed effect of silver species on hydrocarbon pool in ethylene conversion on ZSM-5 by 2D COS FT-IR and 2D COS UV-vis *operando* spectroscopies

Karolina A. Tarach, Agata Kordek, Oliwia Rogala, Gabriela Jajko-Liberka, Małgorzata Smoliło-Utrata, Anna Walczyk, Joaquin Martinez-Triguero, Fernando Rey, and Kinga Góra-Marek

Structural (XRD) and textural (low-temperature N_2 sorption) characterization of studied materials

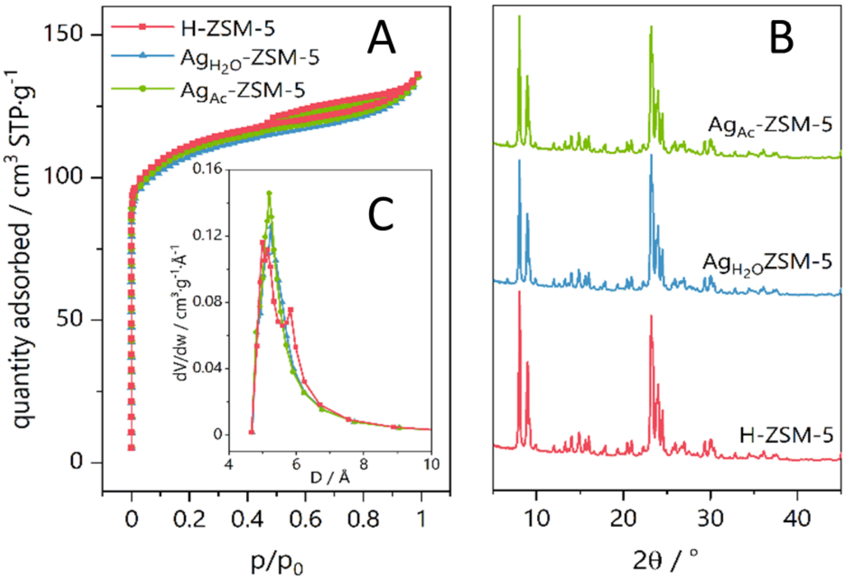

Figure S1 Low-temperature nitrogen physisorption isotherms (A) with Horvath-Kawazoe differential pore volume plot (inset, C) and X-ray powder diffraction patterns (B) for all studied samples.

Table S1 The relative crystallinity and textural properties from low-temperature nitrogen sorption of studied catalysts. Silver ion-exchange procedure with relatively low Ag-content altered neither the specific surface area nor the micropores volume. No Si/Al ratio changes are observed upon silver deposition, regardless of the protocol applied.

**catalyst**	**rel. cryst.[a] %**	**S_{BET} $m^2 \cdot g^{-1}$**	**V_{micro} $cm^3 \cdot g^{-1}$**	**V_{meso} $cm^3 \cdot g^{-1}$**	**Si/Al**	**Ag_{ICP} μmol·g⁻¹**
**H-ZSM-5**	100	423	0.17	0.04	11.5	-
**Ag_{H_2O}-ZSM-5**	95	408	0.16	0.05	11.3	167
**Ag_{Ac}-ZSM-5**	93	415	0.17	0.05	11.2	156

[a] based on XRD studies and calculated as the ratio of the sum of the integral intensity of the most intense reflections within the 2Θ angle range from 21.5° to 25.5° to the sum of integral intensity for native zeolite.

Morphology (TEM) of studied materials

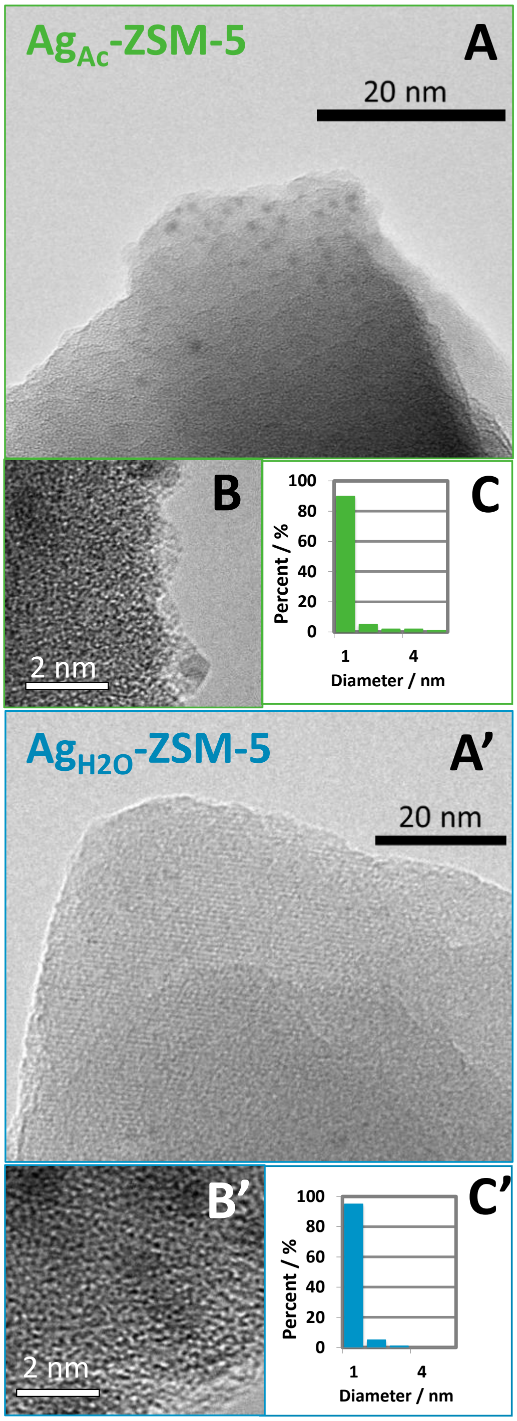

Figure S2 (A, A') TEM images, (B, B') HR-TEM images, and (C, C') silver particle size distribution diagram of Ag_{Ac}-ZSM-5 and Ag_{H_2O}-ZSM-5. The nanoparticles have a very narrow size distribution. The average size of the encapsulated Ag particles was determined to be 2.8 and 2.0 nm for Ag_{Ac}-ZSM-5 and Ag_{H_2O}-ZSM-5, respectively. Scale bars: (A, A') 20 nm and (B, B') 2 nm of grain size.

Method S1: Nature of silver sites (colour of sample vs. dispersion of silver) in studied materials

The Ag_{H_2O}-ZSM-5 was colourless under atmospheric conditions, while the yellowish colour of Ag_{Ac}-ZSM-5 was observed. This has not yet been reported under atmospheric conditions. It suggests that in the Ag^+ coordination sphere, both water and acetone serve as ligands. In the literature, the yellowish colour is discussed in connection with the formation of silver clusters[1], but recently, based on the vacuum-dehydration treatment of Ag-A zeolite under increasing temperature, the yellow-to-red colour was ascribed to the charge-transfer transition from the oxygen atoms of the zeolite lattice to the 5s orbital of silver cations[2]. Fully dehydrated Ag-A zeolites turned colourless again when exposed to humidity. However, the colour of Ag_{Ac}-ZSM-5 persisted. Zeolite Ag_{Ac}-ZSM-5 exhibits a reduced concentration of Ag^+ cations compared to zeolite Ag_{H_2O}-ZSM-5, as indicated by CO sorption studies. Consequently, variations in sample colour are primarily associated with differences in the degree of silver dispersion within both zeolites.

In-situ FT-IR spectroscopy for assessment of organic species decomposition in studied materials

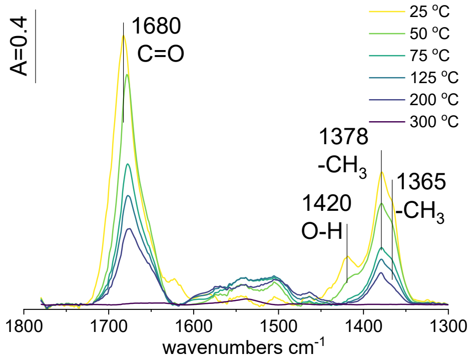

Figure S3 FT-IR spectra collected during thermal treatment of overnight dried (24 h, 100 °C) Ag_{Ac}-ZSM-5-nc under vacuum conditions. The spectra prove the persistence of acetone-derived forms on the catalyst's surface. The acetone-derived species were found on the surface of Ag_{Ac}-ZSM-5-nc. After drying (24 h, 100 °C), the non-calcined sample was heated slowly under a vacuum in an IR cell, and spectra were collected. It was found that the C=O band[3] at 1680 cm^{-1}, representative for acetone bonded to Ag^+ persisted till 200 °C. After calcination (550 °C for 2 h in air atmosphere), these bands were no longer observed[4], confirming the complete removal of all organic species from Ag_{Ac}-ZSM-5. Scale bar: 0.4 a.u bands intensity.

In-situ FT-IR spectroscopy for assessment of Si(OH)Al groups nature in studied materials

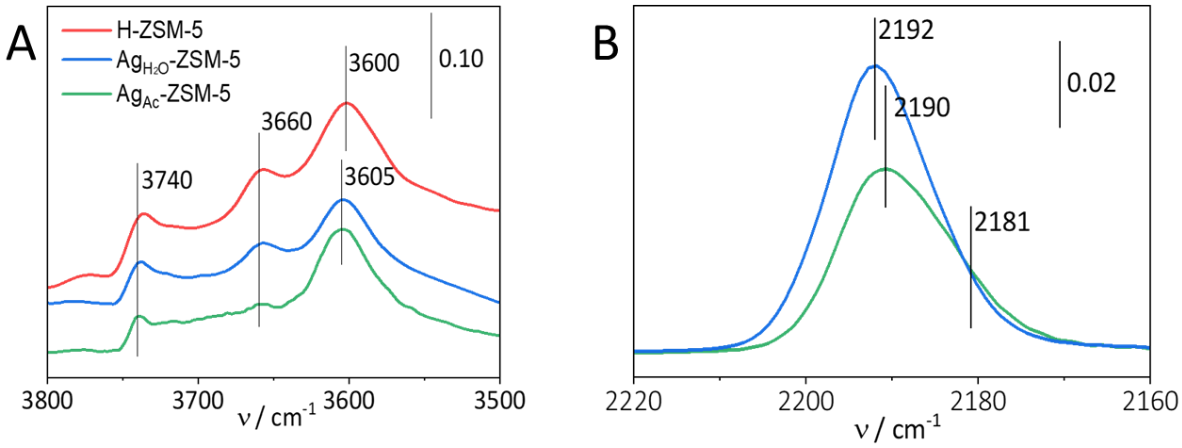

Figure S4 FT-IR spectra of Si(OH)Al groups of activated catalysts (A) and CO adsorbed at RT on the Ag-zeolites surface (B). The Si(OH)Al groups of the highest acidity are eliminated due to their replacement with silver cationic species. The Si(OH)Al groups in Ag-zeolites are therefore characterised by lower strength. Ag_{Ac}-ZSM-5 is characterised by a higher intensity of the bridging groups band than Ag_{H_2O}-ZSM-5. Therefore, a smaller number of silver ions neutralise the negative charge of the zeolite framework, which consequently suggests the presence of silver sites of lower dispersion. The spectra of CO adsorbed on Ag-zeolites display exclusively the bands (2192-2190 cm^{-1}) of monocarbonyls formed on silver cations. The downshift of the Ag^{+}(CO) band signifies a decrease in the electron-acceptor properties of silver cations[5]. At the same time, the reduction in intensity suggests a diminished quantity of silver cations available for interaction with the probe molecule. Scale bars: 0.10 a.u. (A), 0.02 a.u. (B) of bands intensity.

XPS studies of studied materials

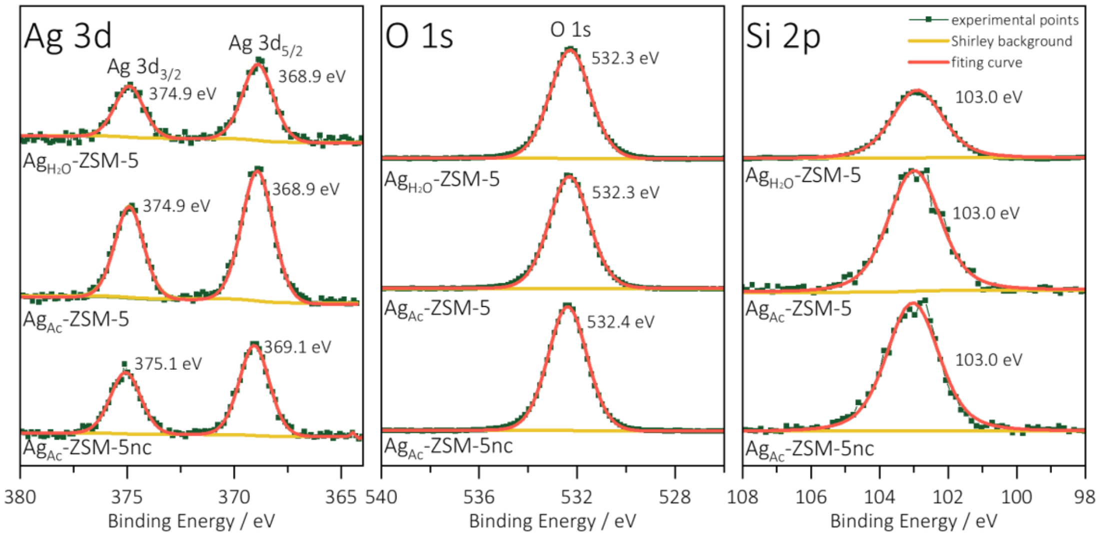

Figure S5 XPS spectra of calcined Ag_{H_2O}-ZSM-5 and Ag_{Ac}-ZSM-5, as well as of non-calcined Ag_{Ac}-ZSM-5-nc samples. The XPS spectra of AgH_2O-ZSM-5, AgAc-ZSM-5, and AgAc-ZSM-5-nc catalysts in the region of Ag 3d present simple doublets with an energy distance of ca. 6.0 eV. The Ag 3d5/2 binding energies equal 368.9-369.1 eV and could be assigned to the Ag^+ cations at the exchange positions[6, 7]. No additional peaks of Ag 3d at lower binding energy values (below 368 eV) were found; thus, the presence of metallic species was excluded. The binding energy positions of Ag 3d are insufficient to perform a comprehensive analysis of the silver oxidation states. It should also be noted that XPS is a surface-sensitive technique, i.e., ca. 95% of the XPS signal originates from the layer with a thickness typically 10-20 Å, depending on the material.

Quantitative FT-IR studies of probe molecules adsorption on the surface of studied materials

Table S2 Chemical composition (Si/Al ratio, Ag content in dehydrated zeolite). The quantitative results of *in situ* FT-IR spectroscopic studies of probe molecule sorption; (Ag^+(CO)) – concentration of Ag sites from CO sorption; BAS, LAS, and BAS+LAS –concentration of Brønsted and Lewis acid sites, and their sum from Py sorption at 170 °C). The strength of Brønsted acid sites from FT-IR thermal desorption studies. Native H-ZSM-5 contains both Brønsted and Lewis acid sites, the latter accounting for only 17% of the total number of acid sites that could be detected using Py. As expected, the Py sorption confirms the lower concentration of BAS after Ag introduction. At the same time, a 4-fold and 2-fold increase in the number of Lewis acid sites is found for Ag_{H_2O}-ZSM-5 and Ag_{Ac}-ZSM-5, respectively. This difference in LAS density between both Ag zeolites further indicates the increased nuclearity of silver clusters in Ag_{Ac}-ZSM-5, which was previously inferred from the variation in Si(OH)Al band intensity and higher BAS concentration for this sample. A decrease in the strength of the Brønsted acid sites was also observed after the introduction of Ag. Carbon monoxide also shows significant differences in silver speciation, despite its significantly lower basicity than Py molecule. This difference in basicity of both probe molecules means that if not sterically constrained, the Py molecule can interact with a larger number of acid sites. The concentration of silver cations (derived from carbon monoxide sorption) was compared to the total number of silver atoms (based on chemical composition) to evaluate the average aggregation of silver species. This quantitative assessment assumes that the IR absorption coefficient of the silver monocarbonyls band is independent of cluster size and that clusters of uniform size are produced.

**catalyst**	**Si/Al**	**Al μmol·g⁻¹**	**Ag μmol·g⁻¹**	**Ag^+(CO) μmol·g⁻¹**	**$Ag^+(CO)/Ag^{ICP}$**	**BAS μmol·g⁻¹**	**LAS μmol·g⁻¹**	**BAS+LAS μmol·g⁻¹**	**Strength BAS**
**HZSM-5**	11.5	1245	-			950	200	1150	0.95
**Ag_{H_2O}-ZSM-5**	11.3	1265	167	97	0.58	750	850	1600	0.80
**Ag_{Ac}-ZSM-5**	11.2	1275	156	57	0.37	860	390	1250	0.78

Operando UV-vis spectroscopic studies of silver speciation before the contact with ethylene

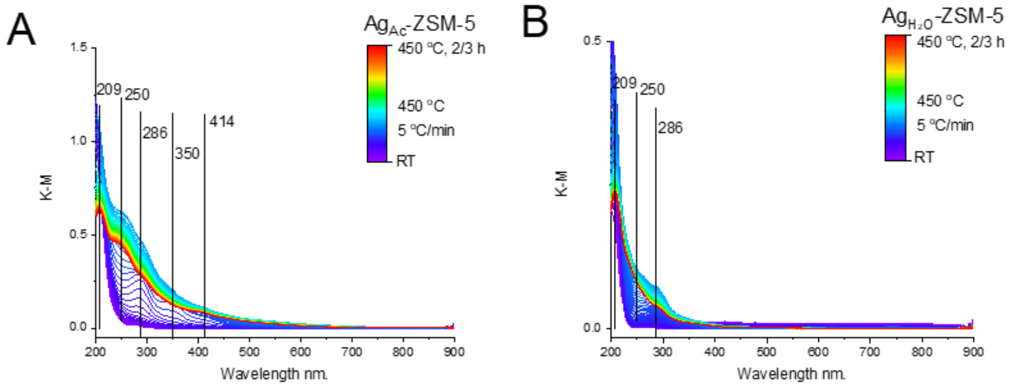

Figure S6 UV-vis spectra registered during (A) Ag_{Ac}-ZSM-5 and (B) Ag_{H_2O}-ZSM-5 sample activation in the flow of synthetic air prior to *operando* studies of ethylene oligomerization. The temperature was gradually raised from room temperature to 450 °C over a period of three hours. Following oxidation pre-treatment, the Ag_{Ac}-ZSM-5 sample demonstrates increased heterogeneity of silver forms, as indicated by the diversity of UV-vis bands, representative of cationic (209 nm), aggregated (250, 286 nm), and nanosized (350, 414 nm) species. The Ag_{H_2O}-ZSM-5 catalyst contains silver mainly as isolated cations Ag^+ (209 nm) with only a minor share of other forms. Scale bars: Temperature (425 °C) and time (3 hours).

Mass spectrometry for ethylene oligomerization

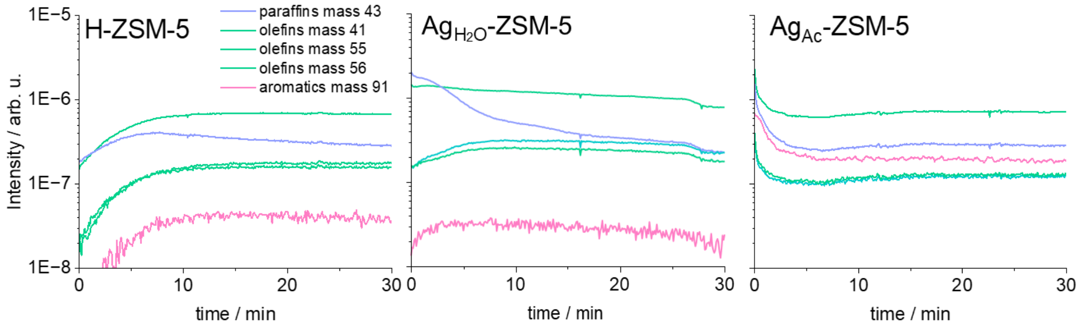

Figure S7 Mass spectrometry signals of ethylene oligomerization products for studied zeolite registered during first 30 min of time on stream in *operando* spectroscopic experiments. These MS data are complementary to chromatographic analyses and UV-vis spectra.

Gas chromatography for ethylene oligomerization

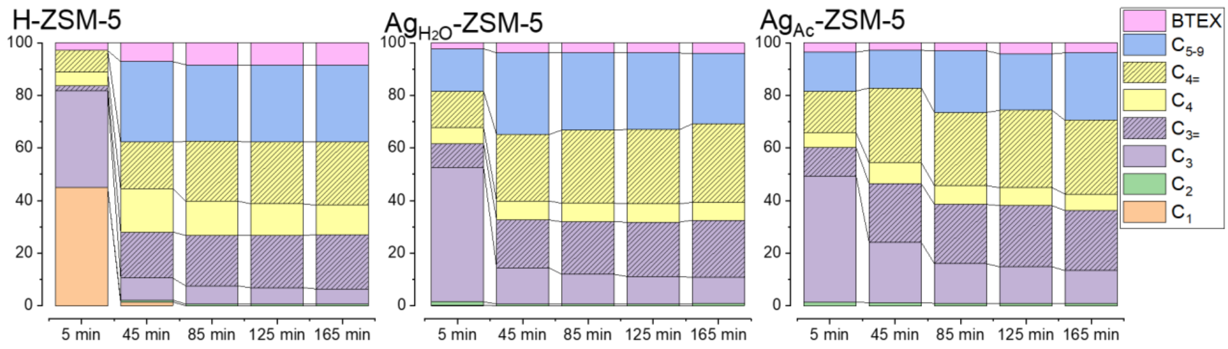

Figure S8 Gas chromatography results of ethylene oligomerization over studied zeolites collected during the reaction course in *operando* spectroscopic experiments.

Critical evaluation of UV-vis and FT-IR bands assignment

Table S3 The species identified in the hydrocarbon pool mechanism and their UV-vis and IR bands assignment.*

	**UV-vis signature / nm**	**IR signature / cm⁻¹**	**Reference**
hexamethylbenzene neutral	204 and 225	1453, 1417, and 1377	8
trienes neutral	240-280	1600	9
hexamethylbenzene protonated	279 and 391		10, 11
1,2,3,4,5-pentamethylcyclopentadiene protonated	299		10
1,2,3,4,5-pentamethylcyclopentadiene neutral	< 270		10, 11
alkyl-substituted benzene neutral	265		This work
1,3-dimethylcyclopentadienium ion	275		10, 11
1-ethyl-3-methylcyclopentadienium ion	278		10, 11
1,3,4-trimethylcyclopentadienium ion	279		10, 11
alkyl-substituted cyclopentenyl cation	285		This work
1,2,3,4-tetramethylcyclopentadienium ion	294		10, 11
1,2,3,4,4-pentamethylcyclopentadienium ion	299		10, 11
methylbenzenium ion	320		10, 11
alkyl-substituted benzenium ion	325		This work
trimethylbenzenium ion	335		12
methyl/ethylbenzenium ions with four alkyl groups	350	1605	10
methylated naphthalene carbocations *or* neutral polycyclic aromatic species	415		10, 11, 13
1,3,5-trimethylbenzene		1505	14
1,2,4-trimethylbenzene		1605	14
polyalkyl-substituted cyclopentenyl cations with a proton in the C-2 position and with ten C atoms		1505 ($\nu_{C=C}$) and 1460 (δ_{CH_2})	12
1,2,4,5-tetramethylbenzene neutral		1507, 1464, 1453 and 1383, 1367	15
pentamethylbenzene		a doublet at ca. 1450 and 1580-1540	16
acyclic monoenyl cation (e.g. 2,4-dimethylpentenyl cation)		1545	8
dienyl cations	398	1576	8
alkylnaphthalenes cations	395, 410-415	1540	10, 13, 17

*The recent literature provides a strong background in identifying the range of possible intermediate species in hydrocarbon conversion over zeolites. The ethylene-to-propylene (ETP) process has been reported to operate via the hydrocarbon pool mechanism, involving species analogous to those found in alcohols-to-olefins conversion[18,19]. The propene was also identified as an essential intermediate in the MTH (methanol-to-hydrocarbons) process, as cyclopentenyl cations formation was postulated during propene oligomerization[20]. Alkyl-substituted benzenium and cyclopentenyl cations formed over acidic zeolites during the hydrocarbon pool are well established in the literature[21]. Detailed studies of UV-vis spectra of benzenium and cyclopentenyl cations formation during methanol conversion are provided by Wulfers and Jentoft[10]. The 279 and 391 nm bands identify the protonated hexamethylbenzene in H-BEA zeolite, while neutral species exhibit the most pronounced bands at 204 and 225 nm. Additionally, an increasing number of methyl groups on the methylbenzenium ion results in a bathochromic shift for the absorption band at wavelengths surpassing 320 nm[10,11]. The protonated 1,2,3,4,5-pentamethylcyclopentadiene on H-MOR is identified by the UV-vis band at 299 nm[10], whereas neutral species exhibit bands below 270 nm with significantly lesser intensity. Distinct UV-vis bands are used to characterise cyclopentenyl cations family: 1,3-dimethylcyclopentadienium (275 nm), 1-ethyl-3-methylcyclopentadienium (278 nm) 1,3,4-trimethylcyclopentadienium (279 nm), 1,2,3,4-tetramethyl-cyclopentadienium (294 nm), and 1,2,3,4,4-pentamethylcyclopentadienium (299 nm) ions[10,11]. The bands around 415 nm can be ascribed to species with two or more fused aromatic rings, e.g., methylated naphthalene carbocations or neutral polycyclic aromatic species[10,11,13]. Using solid-state NMR spectroscopy and GC-MS analysis, cyclopentenyl cations were identified as active intermediates during propane aromatisation on zeolite Ga/ZSM-5[22]. The formation of cyclopentenyl cations in the initial stage leads to an autocatalytic reaction, which is well-recognised in methanol conversion to hydrocarbons over acidic zeolites.

UV-Vis studies for reduced ethylene loading

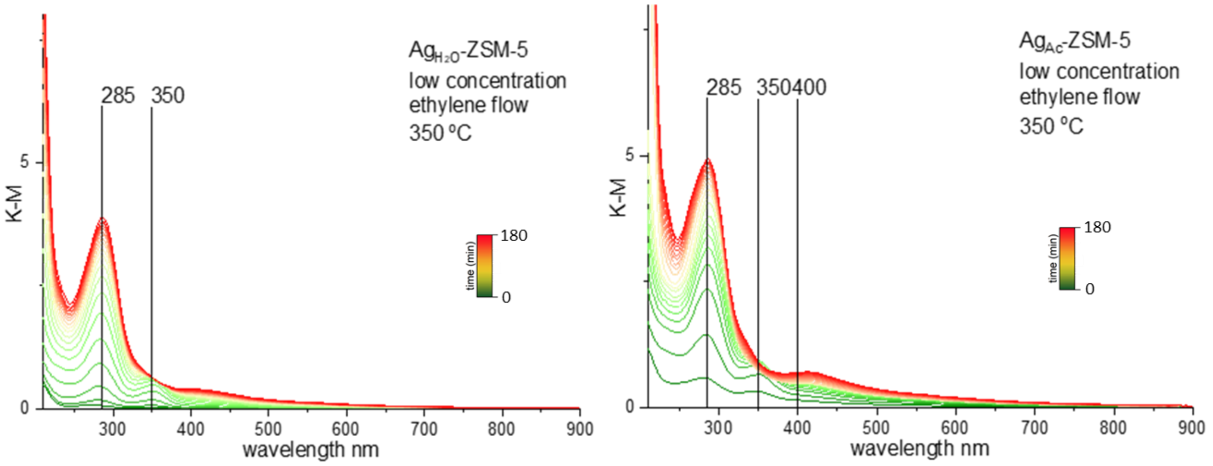

Figure S9 UV-vis spectra registered during ethylene oligomerization over silver-loaded zeolite when the ethylene concentration in the stream was reduced four times (0.5 ml • min^{-1} of ethylene, 23.5 ml • min^{-1} of nitrogen). The other experimental conditions remained unaltered compared to experiments with higher ethylene flow. Similarly to H-ZSM-5, alkyl-substituted cyclopentenyl cations (285 nm) are the main intermediate emerging during the induction period. The protonated acyclic alkyl-substituted trienes, methyl/ethylbenzenium ions with four alkyl groups (350 nm) are formed to a minor extent. The methylated naphthalene carbocations (395 nm) are not present. In Ag$_{Ac}$-ZSM-5, the methyl/ethylbenzenium ions (350 nm), present in the induction period, are consumed in favour of the alkyl-substituted cyclopentenyl cations (285 nm). Similarly as for higher ethylene loading, the maximum intensity of the 285 nm band is attained more rapidly for the Ag$_{Ac}$-ZSM-5 than the Ag$_{H_2O}$-ZSM-5 catalyst. Ag-zeolites also differ in the rate of formation of naphthalenic species. For Ag$_{Ac}$-ZSM-5, the band of naphthalenic species at 415 nm appears immediately after ethylene introduction. All zeolites under study exhibit intensive UV-vis bands below 260 nm, associated with the accumulation of neutral conjugated alkenes and/or aromatics. Scale bars: Time of the reaction (min)

Ethylene oligomerization: the hydroxyls groups tracked by FT-IR

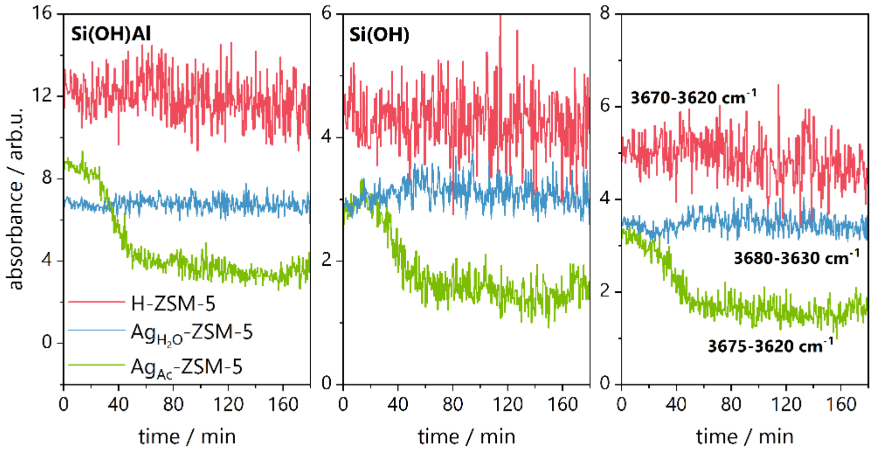

Figure S10 Traces of integral intensity changes of IR bands representative for Si(OH)Al (3620 – 3590 cm^{-1}), Si(OH) (3750 – 3700 cm^{-1}), and Al(OH) (3675 – 3625 cm^{-1}) group regions taken from operando spectroscopic measurement of ethylene oligomerization. The analysis of FT-IR data started with examining time changes in the intensity of hydroxyl bands (3800 – 3500 cm^{-1}), which serve as indicators of product accumulation on the catalyst surface. In the Ag$_{Ac}$-ZSM-5 sample, hydroxyls of all types are fully occupied after 60 minutes of reaction, as evidenced by the decrease in their integral intensity; the most pronounced effect is observed for bridging Si(OH)Al groups. For H-ZSM-5 and Ag$_{H_2O}$-ZSM-5, the hydroxyl groups are less affected or fully preserved throughout FT-IR operando experiments.

Operando FT-IR spectroscopic measurement of ethylene oligomerization; time-dependent evolution of critical products

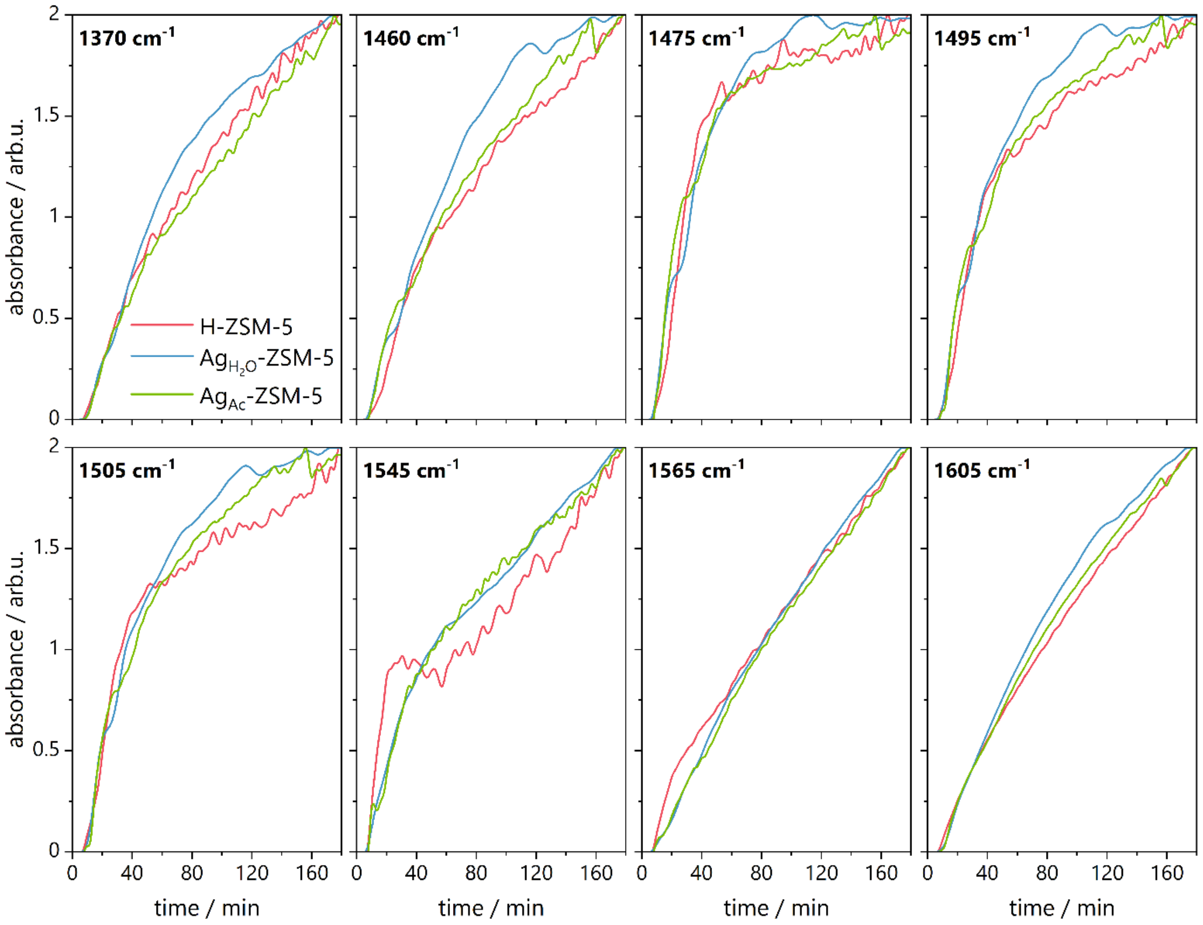

Figure S11 Normalised traces of specific IR band intensities (stretching C=C and deformation C-H vibrations, 1700 – 1300 cm^{-1}) taken from operando spectroscopic measurement of ethylene oligomerization. The most pronounced bands at 1600, 1505, and 1370 cm^{-1} found during the very first few minutes of ethylene introduction suggest the formation of neutral conjugated alkenes (1650 – 1600 cm^{-1})[23], neutral polymethylbenzenes, more specifically the 1605 cm^{-1} band is suggested for 1,3,5-trimethylbenzene while the band at 1505 cm^{-1} is due to 1,2,4-trimethylbenzene[14]. Within the next few minutes of reaction, the pronounced bands at ca. 1600, 1500, and 1370 cm^{-1} are evident on FT-IR spectra. Nevertheless, the band at 1505 cm^{-1} rises more rapidly and, after 60 minutes, attains a plateau, while the band at 1600 cm^{-1} persists in its constant increase throughout the entire reaction course. Therefore, species other than neutral aromatics must account for forming the 1505 cm^{-1} band. The increased concentration of olefins in the outflow stream following the induction phase (60-180 min) further corroborates this observation. This is also confirmed by the higher amount of olefins in the outlet stream after the induction period (60-180 min). Therefore, the IR band at 1505 cm^{-1} (4C=C) with a shoulder at 1460 cm^{-1} (4CH_2) is assigned to an alkyl-substituted cyclopentenyl cation[10,24].

2D COS synchronous and asynchronous analysis for operando FT-IR and UV-vis spectroscopic data

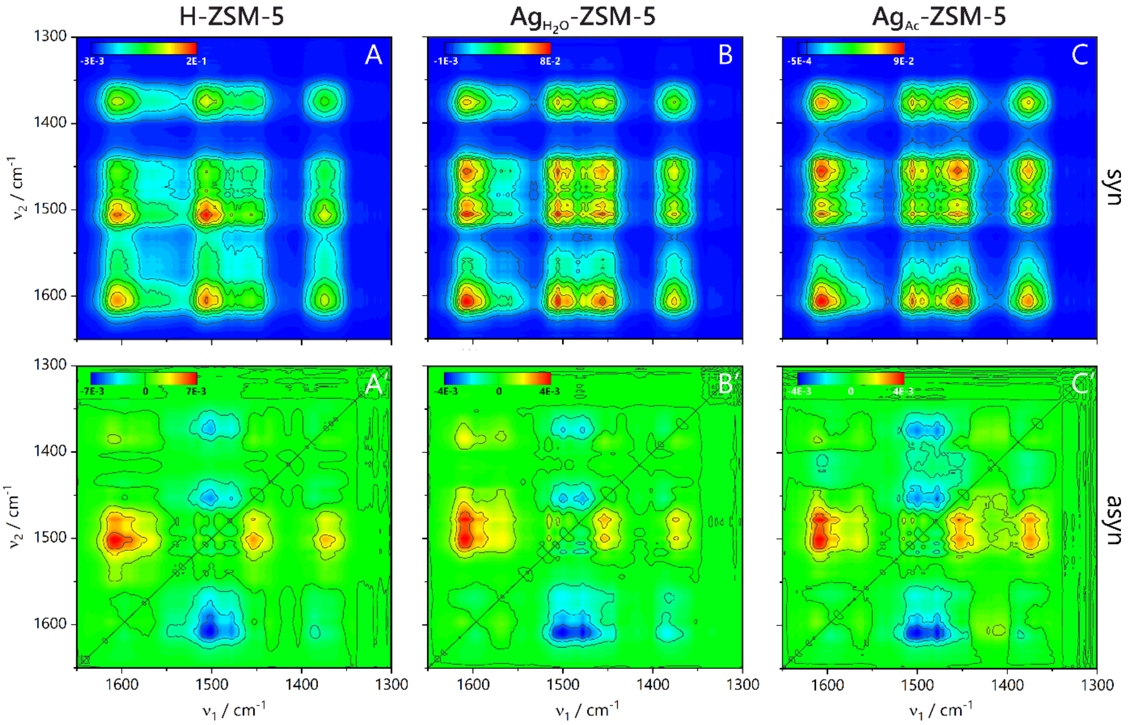

Figure S12 2D COS synchronous (A, B, C) and asynchronous (A', B', C') maps of FT-IR spectra in the region 1650 – 1300 cm^{-1} obtained during ethylene oligomerization (180 min) over H-ZSM-5 (A, A'), Ag$_{H_2O}$-ZSM-5 (B, B'), and Ag$_{Ac}$-ZSM-5 (C, C'). A significant number of asynchronous correlation peaks facilitates the identification of a clear sequence of events for the Ag$_{Ac}$-ZSM-5 sample. In contrast, the 2D COS asynchronous maps for Ag$_{H_2O}$- and H-ZSM-5 are less detailed. For all samples, the bands located at ca. 1605, 1600, and 1590 cm^{-1} show changes in intensity prior to those at lower wavenumbers, but the representative positive cross-peaks on 2D COS async-maps are the most evident for the Ag$_{Ac}$-ZSM-5 sample. In all samples, the negative cross-peaks for bands located in the range 1505 – 1465 cm^{-1} indicate the increase of these bands before those in lower wavenumber regions: 1465 – 1440 cm^{-1} and 1385 – 1355 cm^{-1}. The 2D COS asynchronous analysis of spectra obtained over an extended reaction time of 180 min points to enhancing the positive cross-peaks while the negative ones become less negative. Thus, the preference for forming compounds identified by the bands at 1605, 1600 and 1590 cm^{-1} becomes enhanced later in the reaction (60–180 min). The visibility of cross-peaks for other bands diminishes, compromising the inference of the order of appearance of the associated hydrocarbon species. Scale bars: correlation intensity (a.u.).

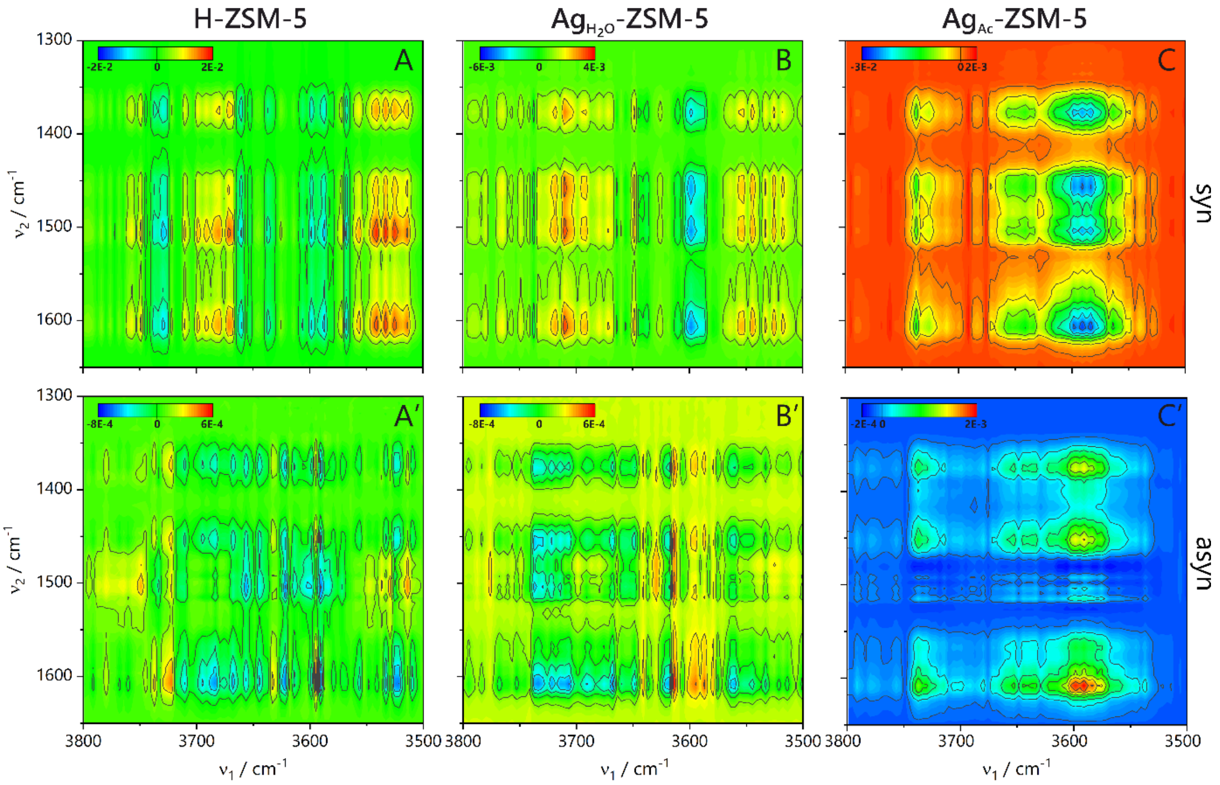

Figure S13 2D COS synchronous (A, B, C) and asynchronous (A’, B’, C’) maps of FT-IR spectra in mixed region 1650 – 1300 cm^{-1} x 3800 – 3500 cm^{-1} obtained during ethylene oligomerization (180 min) over H-ZSM-5 (A, A’), Ag_{H_2O}-ZSM-5 (B, B’) and Ag_{Ac}-ZSM-5 (C, C’). In the 2D COS map for Ag_{Ac}-ZSM-5, the highest correlation is found between the bands located at ca. 1600&1450 cm^{-1} and the band representative for Si(OH)Al groups (3610 cm^{-1}). Similar changes, but at a limited level, are found in 2D COS maps of the H-ZSM-5 and Ag_{H_2O}-ZSM-5. According to these correlations, the Si(OH)Al band undergoes the intensity change first, followed by the bands at ca. 1600 cm^{-1}. The decreasing intensity of Si(OH)Al groups in Ag_{Ac}-ZSM-5 indicates that the proton from the Si(OH)Al groups is being accepted by intermediate products created during the ethylene reaction. This effect cannot be ascribed to the coke deposit formation since there was no discernible decrease in activity throughout the catalytic tests or operando spectroscopic investigations. Therefore, active site poisoning and deactivation are ruled out. Accordingly, the intensity of Si(OH)Al groups decreases after the formation of neutral polyenes and the $Ag(C_2H_4)^+$ complexes. The 2D COS maps do not allow concluding the change order between the bands of the Si(OH)Al groups and cyclopentenyl cations. Scale bars: correlation intensity (a.u.).

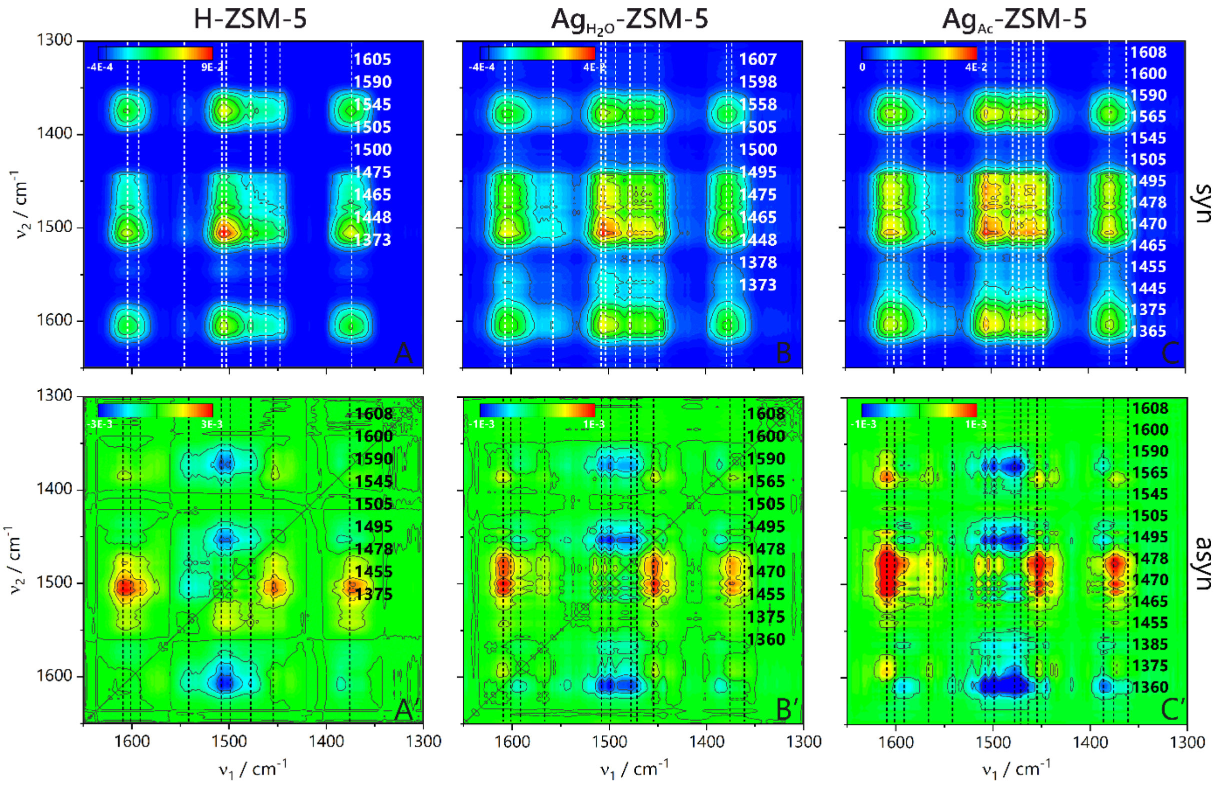

Figure S14 2D COS synchronous (A, B, C) and asynchronous (A', B', C') maps for FT-IR spectra in the region 1650 – 1300 cm^{-1} for ethylene oligomerization (first 60 min) over H-ZSM-5 (A, A'), Ag$_{H_2O}$-ZSM-5 (B, B'), and Ag$_{Ac}$-ZSM-5 (C, C'). Important insight was gained by analysing the spectra collected during the first 60 minutes in region 1650 – 1300 cm^{-1}. In the 1650 – 1300 cm^{-1} region of the synchronous maps, only positive peaks are observed for all samples studied, indicating the species accumulation on the catalysts as identified by the wavenumber of the respective cross peaks. In the initial 60 minutes of the reaction over H-ZSM-5, significant alterations are observed for the bands around 1505 cm^{-1}. At longer reaction times, the intensities of auto-peaks located at ca. 1600 cm^{-1} and at ca. 1450 cm^{-1} are enhanced (Figure S12). In the synchronous maps of silver-containing zeolites, the intensive auto-peaks are located at lower wavenumbers, and this effect is amplified with increased reaction time. The Ag$_{Ac}$-ZSM-5 sample exhibits the highest intensity auto-peaks at 1455 cm^{-1} and approximately 1600 cm^{-1} on the sync-map after 180 minutes of reaction (Figure S12). Additionally, in silver-containing samples, all peaks on the maps exhibit increased broadness and asymmetry, accompanied by significant tailing at lower wavenumbers. Scale bars: correlation intensity (a.u.).

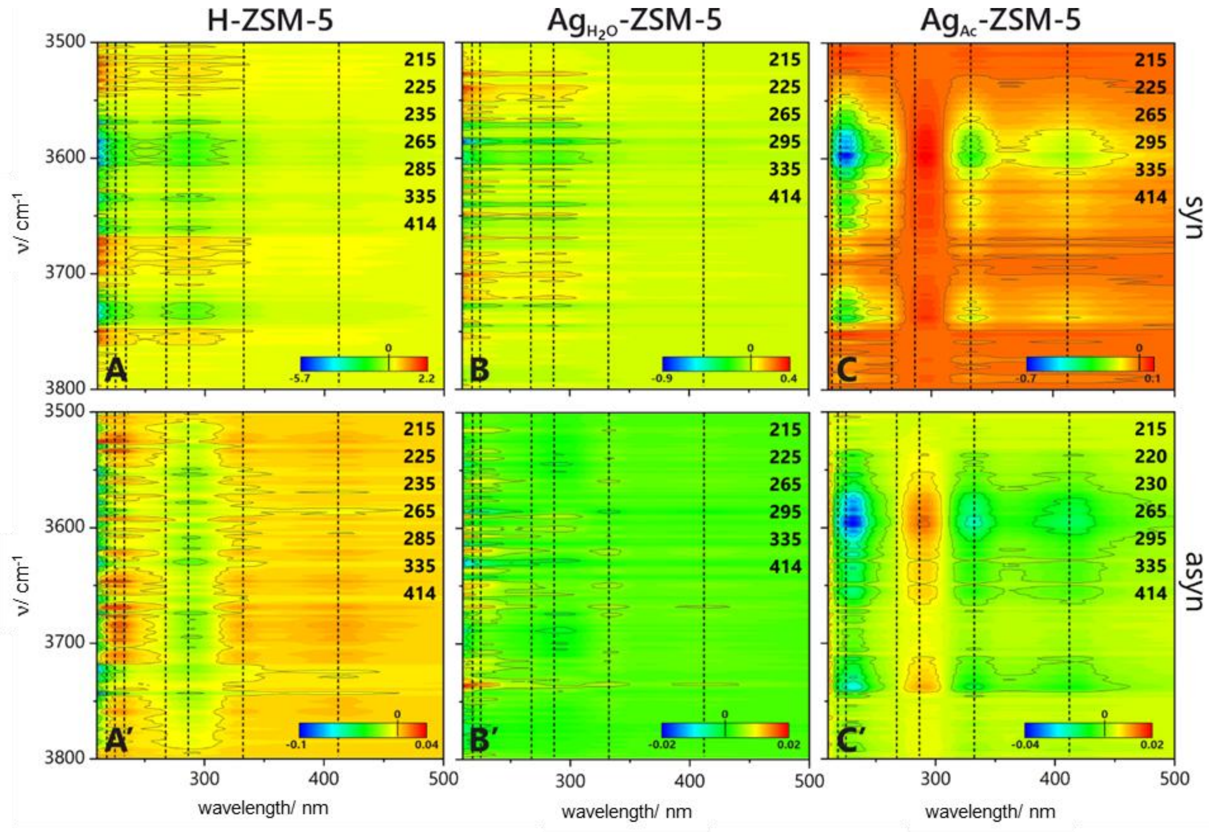

Figure S15 2D COS synchronous (A, B, C) and asynchronous (A', B', C') heterospectral maps of FT-IR and UV-vis regions 3800 – 3500 cm^{-1} x 200 – 500 nm obtained during ethylene oligomerization (180 min) over H-ZSM-5 (A, A'), Ag_{H_2O}-ZSM-5 (B, B') and Ag_{Ac}-ZSM-5 (C, C'). In the presented synchronous maps, limited development of correlation peaks is visible. Only weak negative correlations between the bands at ca. 3740 (Si(OH) groups) and 3610 cm^{-1} (Si(OH)Al groups) and the bands below 300 nm are found. In 2D asynchronous maps, negative or no correlations are present at the same location. The species identified by the bands at 215, 265, and 285 nm are therefore accumulated much faster than the Si(OH)Al groups (3610 cm^{-1}), and Si-OH (3740 cm^{-1}) are consumed. The 2D sync-map for the Ag_{Ac}-ZSM-5 demonstrates the significant decrease of the hydroxyl bands (3600–3500 cm^{-1}) along with the production of intermediates, as evidenced by the increase of all UV-vis bands. The only exception is the 295 nm band, which intensity decreases. For the Ag_{Ac}-ZSM-5 sample, the highest correlation level is found between the band of Si(OH)Al groups (3610 cm^{-1}) and the 215, 220, and 295 nm bands. All intensity changes of bands in the UV-vis region (215, 225, 265, 295, 335, and 414 nm) appear before those in the IR region (3620 – 3590 cm^{-1}, 3675 – 3625 cm^{-1} and 3750 – 3700 cm^{-1}), according to the 2D asynchronous map. This set of correlations confirms that the accumulation of neutral olefins, aromatics (<235 nm, 265 nm), alkyl-substituted cyclopentenyl cations (285 nm), as well as alkyl-substituted benzenium ions and naphthalenes (330 and 415 nm), precedes the consumption of all types of O-H group bands in the IR range. This effect is the most pronounced for the Ag_{Ac}-ZSM-5 sample. Scale bars: correlation intensity (a.u.).

Insight into coke species formed over protonic and silver-loaded zeolites by XPS and GC-MS

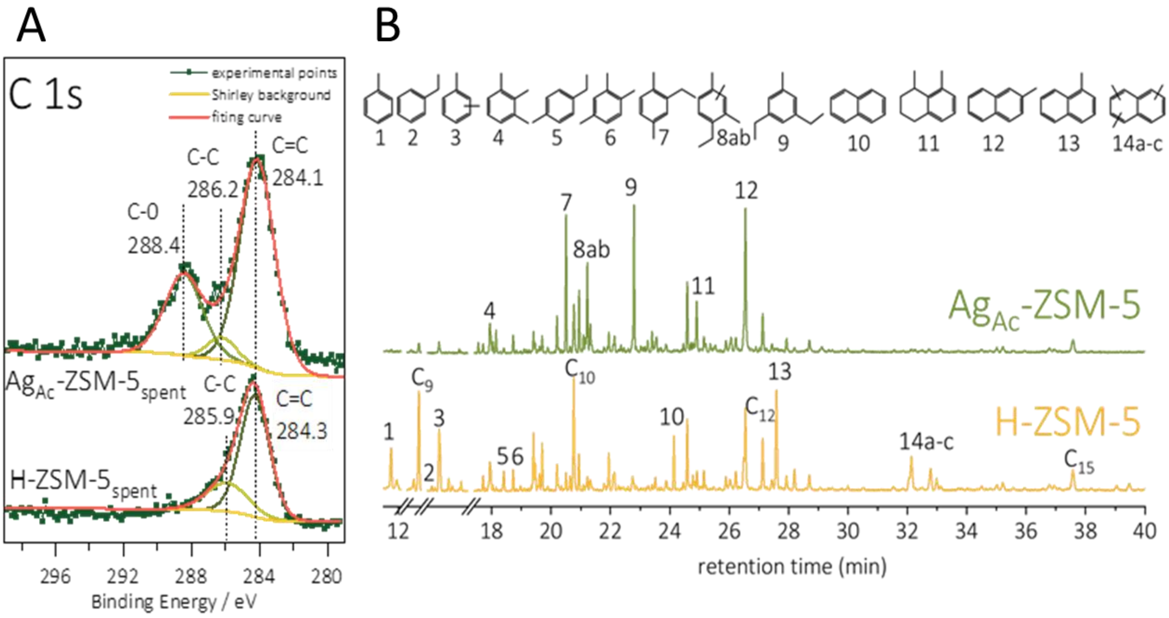

Figure S16 XPS spectra of C1s (A) and GC-MS results of extracted coke (B) from spent H-ZSM-5 and Ag_{Ac}-ZSM-5 catalysts. The XPS of C1s spectra for spent H-ZSM-5 and Ag_{Ac}-ZSM-5 catalysts showed the presence of C1s components such as C=C (284.1 - 284.3 eV), C–C (285.9–286.2 eV), and, selectively for AgAc-ZSM-5, the C–O (288.4 eV) was found. The oxygen-containing bonds might be formed due to defect sites in polyaromatic hydrocarbon coke, which provide suitable anchoring sites for these functionalities. Nevertheless, the presence of the silver cations might also influence the coke speciation by providing the redox centres for partial oxidation at the temperature of XPS measurement of surface-located coke species with trace oxygen adsorbed. The GC-MS analysis of extracted coke species showed another clear distinction between the protonic and silver-containing samples. Firstly, the coke produced over protonic zeolite is more heterogeneous and contains more aliphatic, monoaromatic, and polyaromatic species than the coke from Ag_{Ac}-ZSM-5 spent catalyst. The coke of H-ZSM-5 zeolite contains fewer alkyl-substituted aromatic species compared to the coke of the Ag_{Ac}-ZSM-5 sample.

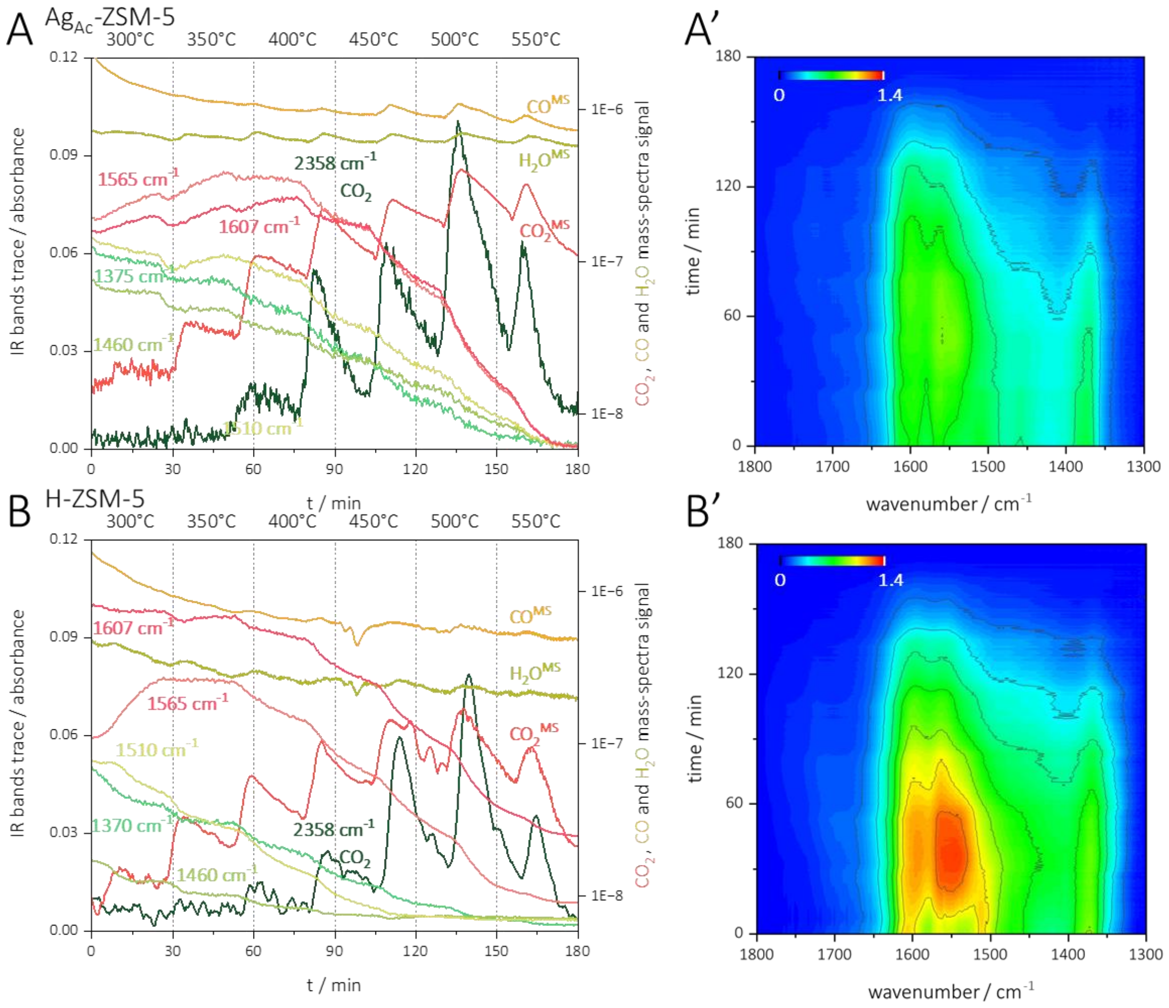

Figure S17 Results of IR-TPO experiments of coke oxidation after operando FT-IR-GC-MS studies of ethylene oligomerization. (A, B) Alteration of IR bands located at 1375 (δCH_3), 1460 (δCH_2), 1510, 1565, 1607 and 2358 cm^{-1} (CO_2) and mass spectrum signal of CO_2 (m/z = 44), CO (m/z =28), and H_2O (m/z = 18) in outlet gases, together with (A', B') the top-down projections of FT-IR spectra registered during IR-TPO experiments of coke oxidation over Ag_{Ac}-ZSM-5 (A, A') and H-ZSM-5 (B, B') catalysts. The mass spectrometry analysis of the signal for oxidation products demonstrates that higher amounts of CO_2, CO and H_2O were detected for Ag_{Ac}-ZSM-5; it might be inferred that this sample likely produces greater quantities of soft coke or that the presence of silver enhances its oxidation regardless of its composition. The H-ZSM-5 zeolite is enriched in hard coke species, requiring temperatures over 550 °C for oxidation. The FT-IR spectra (A', B') and traces of bands (A, B) recorded during coke oxidation demonstrate that at the very first step, the -CH_3 and -CH_2 species are oxidised, and this is followed by aromatic species oxidation. The band at 1565 cm^{-1} again increases its intensity, even during oxidation, due to some aromatisation progress. Scale bars: (A', B') bands intensity (a.u.).

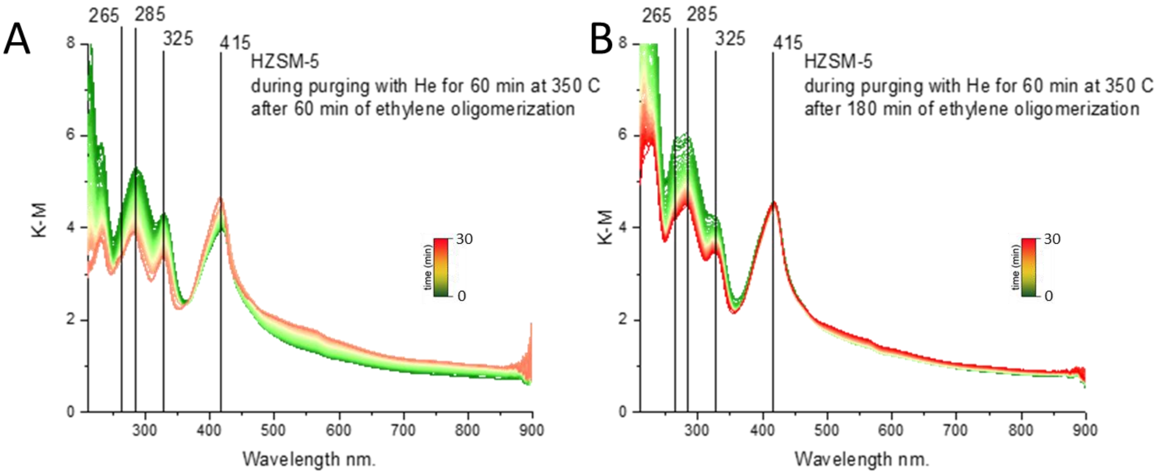

Figure S18 UV-vis spectra registered during purging the H-ZSM-5 surface with inert gas after 60 (A) and 180 min (B) ethylene oligomerization at 350 °C. Scale bars: 30 min of the reactions time.

SI 1. SUPPLEMENTAL REFERENCES

1. Gellens, L. R.; Mortier, W. J.; Uytterhoeven, J. B., On the nature of the charged silver clusters in zeolites of type A, X and Y. Zeolites 1981, 1 (1), 11-18. https://doi.org/10.1016/S0144-2449(81)80035-8

2. Seifert, R.; Kunzmann, A.; Calzaferri, G., The Yellow Color of Silver-Containing Zeolite A. Angew Chem Int Ed Engl 1998, 37 (11), 1521-1524. https://doi.org/10.1002/(SICI)1521-3773(19980619)37:11<1521::AID-ANIE1521>3.0.CO;2-V

3. Kukulska-Zając, E.; Datka, J., The IR studies of the interaction of organic molecules with Ag+ ions in zeolites. Microporous and Mesoporous Materials 2008, 109 (1), 49-57. https://doi.org/10.1016/j.micromeso.2007.04.030

4. Kubelková, L.; Nováková, J., Temperature-programmed desorption and conversion of acetone and diethyl ketone preadsorbed on HZSM-5. Zeolites 1991, 11 (8), 822-826. https://doi.org/10.1016/S0144-2449(05)80062-4

5. Lashchinskaya, Z. N.; Gabrienko, A. A.; Prosvirin, I. P.; Toktarev, A. V.; Stepanov, A. G., Effect of Silver Cations on Propene Aromatization on H-ZSM-5 Zeolite Investigated by 13C MAS NMR and FTIR Spectroscopy. ACS Catalysis 2023, 13 (15), 10248-10260. https://doi.org/10.1021/acscatal.3c01591

6. Fonseca, A. M.; Neves, I. C., Study of silver species stabilized in different microporous zeolites. Microporous Mesoporous Mater. 2013, 181, 83-87. https://doi.org/10.1016/j.micromeso.2013.07.018

7. Ju, W.-S.; Matsuoka, M.; Iino, K.; Yamashita, H.; Anpo, M., The Local Structures of Silver(I) Ion Catalysts Anchored within Zeolite Cavities and Their Photocatalytic Reactivities for the Elimination of N2O into N2 and O2. J. Phys. Chem. B 2004, 108 (7), 2128-2133. https://doi.org/10.1021/jp0302919

8. Manookian, B.; Hernandez, E. D.; Baer, M. D.; Mundy, C. J.; Jentoft, F. C.; Auerbach, S. M., Experimental and DFT Calculated IR Spectra of Guests in Zeolites: Acyclic Olefins and Host–Guest Interactions. The Journal of Physical Chemistry C 2020, 124 (19), 10561-10572. https://doi.org/10.1021/acs.jpcc.0c01225

9. Lopes, C. W.; Martinez-Ortigosa, J.; Góra-Marek, K.; Tarach, K.; Vidal-Moya, J. A.; Palomares, A. E.; Agostini, G.; Blasco, T.; Rey, F., Zeolite-driven Ag species during redox treatments and catalytic implications for SCO of NH3. Journal of Materials Chemistry A 2021, 9 (48), 27448-27458. https://doi.org/10.1039/D1TA09625G

10. Wulfers, M.; Jentoft, F., The Role of Cyclopentadienium Ions in Methanol-to-Hydrocarbons Chemistry. ACS Catalysis 2014, 4, 3521-3532. https://doi.org/10.1021/cs500722m

11. Van Speybroeck, V.; Hemelsoet, K.; De Wispelaere, K.; Qian, Q.; Van der Mynsbrugge, J.; De Sterck, B.; Weckhuysen, B. M.; Waroquier, M., Mechanistic Studies on Chabazite-Type Methanol-to-Olefin Catalysts: Insights from Time-Resolved UV/Vis Microspectroscopy Combined with Theoretical Simulations. ChemCatChem 2013, 5 (1), 173-184. https://doi.org/10.1002/cctc.201200580

12 . Li, N.; Huang, B.; Dong, X.; Luo, J.; Wang, Y.; Wang, H.; Miao, D.; Pan, Y.; Jiao, F.; Xiao, J.; Qu, Z., Bifunctional zeolites-silver catalyst enabled tandem oxidation of formaldehyde at low temperatures. Nature Communications 2022, 13 (1), 2209. https://doi.org/10.1038/s41467-022-29936-8

13. Qian, Q.; Vogt, C.; Mokhtar, M.; Asiri, A. M.; Al-Thabaiti, S. A.; Basahel, S. N.; Ruiz-Martínez, J.; Weckhuysen, B. M., Combined Operando UV/Vis/IR Spectroscopy Reveals the Role of Methoxy and Aromatic Species during the Methanol-to-Olefins Reaction over H-SAPO-34. ChemCatChem 2014, 6 (12), 3396-3408. https://doi.org/10.1002/cctc.201402714

14. Gołąbek, K.; Tarach, K. A.; Góra-Marek, K., Standard and rapid scan infrared spectroscopic studies of o-xylene transformations in terms of pore arrangement of 10-ring zeolites – 2D COS analysis. Dalton Transactions 2017, 46 (30), 9934-9950. https://doi.org/10.1039/C7DT00644F

15. Bjørgen, M.; Bonino, F.; Arstad, B.; Kolboe, S.; Lillerud, K.-P.; Zecchina, A.; Bordiga, S., Persistent Methylbenzenium Ions in Protonated Zeolites: The Required Proton Affinity of the Guest Hydrocarbon. ChemPhysChem 2005, 6 (2), 232-235. https://doi.org/10.1002/cphc.200400422

16. Bjørgen, M.; Bonino, F.; Kolboe, S.; Lillerud, K.-P.; Zecchina, A.; Bordiga, S., Spectroscopic Evidence for a Persistent Benzenium Cation in Zeolite H-Beta. Journal of the American Chemical Society 2003, 125 (51), 15863-15868. https://doi.org/10.1021/ja037073d

17. Karge, H. G.; Nießen, W.; Bludau, H., In-situ FTIR measurements of diffusion in coking zeolite catalysts. Applied Catalysis A: General 1996, 146 (2), 339-349. https://doi.org/10.1016/S0926-860X(96)00175-5

18. Lee, K.; Cha, S. H.; Hong, S. B., MSE-Type Zeolites: A Promising Catalyst for the Conversion of Ethene to Propene. ACS Catalysis 2016, 6 (6), 3870-3874. https://doi.org/10.1021/acscatal.6b01057

19. Lee, K.; Hong, S. B., Hydrocarbon Pool Mechanism of the Zeolite-Catalyzed Conversion of Ethene to Propene. ACS Catalysis 2019, 9 (12), 10640-10648. https://doi.org/10.1021/acscatal.9b03434

20. Hawkins, A. P.; Zachariou, A.; Parker, S. F.; Collier, P.; Howe, R. F.; Lennon, D., Studies of propene conversion over H-ZSM-5 demonstrate the importance of propene as an intermediate in methanol-to-hydrocarbons chemistry. Catalysis Science & Technology 2021, 11 (8), 2924-2938. https://doi.org/10.1039/D1CY00048A

21. Olsbye, U.; Svelle, S.; Bjørgen, M.; Beato, P.; Janssens, T. V. W.; Joensen, F.; Bordiga, S.; Lillerud, K. P., Conversion of Methanol to Hydrocarbons: How Zeolite Cavity and Pore Size Controls Product Selectivity. Angewandte Chemie International Edition 2012, 51 (24), 5810-5831. https://doi.org/10.1002/anie.201103657

22. Wang, C.; Zhao, X.; Hu, M.; Qi, G.; Wang, Q.; Li, S.; Xu, J.; Deng, F., Unraveling Hydrocarbon Pool Boosted Propane Aromatization on Gallium/ZSM-5 Zeolite by Solid-State Nuclear Magnetic Resonance Spectroscopy. Angew Chem Int Ed Engl 2021, 60 (44), 23630-23634. https://doi.org/10.1002/anie.202111111

23. Ganjkhanlou, Y.; Berlier, G.; Groppo, E.; Borfecchia, E.; Bordiga, S., In Situ Investigation of the Deactivation Mechanism in Ni-ZSM5 During Ethylene Oligomerization. Topics in Catalysis 2017, 60 (19), 1664-1672. https://doi.org/10.1007/s11244-017-0845-6

24. Hernandez, E. D.; Manookian, B.; Auerbach, S. M.; Jentoft, F. C., Shape-Selective Synthesis of Alkylcyclopentenyl Cations in Zeolites and Spectroscopic Distinction of Constitutional Isomers. ACS Catalysis 2021, 11 (21), 12893-12914. https://doi.org/10.1021/acscatal.1c03039
